# Developing a digital intervention for cancer survivors: an evidence-, theory- and person-based approach

**DOI:** 10.1038/s41746-019-0163-4

**Published:** 2019-09-02

**Authors:** Katherine Bradbury, Mary Steele, Teresa Corbett, Adam W. A. Geraghty, Adele Krusche, Elena Heber, Steph Easton, Tara Cheetham-Blake, Joanna Slodkowska-Barabasz, Andre Matthias Müller, Kirsten Smith, Laura J. Wilde, Liz Payne, Karmpaul Singh, Roger Bacon, Tamsin Burford, Kevin Summers, Lesley Turner, Alison Richardson, Eila Watson, Claire Foster, Paul Little, Lucy Yardley

**Affiliations:** 1 School of Psychology, University of Southampton, Building 44, Highfield,, Southampton, SO171BJ UK; 2 NIHR Southampton Biomedical Research Centre, Southampton Centre for Biomedical Research, MP 218, Southampton General Hospital, Tremona Road, Southampton, SO16 6YD UK; 3 Health Sciences, University of Southampton, Building 67, Highfield, Southampton, SO17 1BJ UK; 4 Primary Care and Population Sciences, University of Southampton, Aldermoor Health Centre, Aldermoor Close, Southampton, SO16 5ST UK; 5 GET.ON Institut für Online Gesundheitstrainings Gmb, Stellv. Geschäftsführung, GET.ON Institut für Online Gesundheitstrainings GmbH, Rothenbaumchaussee 209, 20149 Hamburg, Germany; 6 National University of Singapore, Saw Swee Hock School of Public Health, Tahir Foundation Building, 12 Science Drive 2, #10-01 Singapore, Singapore; 7 Centre for Sport & Exercise Sciences, University of Malaya, Kuala Lumpur, Malaysia; 8 Centre for Innovative Research Across the Life Course, Faculty of Health and Life Sciences, Coventry University, Coventry, UK; 9 Department of Psychology, University of Calgary, Administration Building, Room 24, 2500 University Drive NW, Calgary, AB T2N 1N4 Canada; 10 PPI Representatives, School of Psychology, University of Southampton, Building 44, Highfield, Southampton, SO171BJ UK; 11 School of Nursing and Midwifery, Faculty of Health and Life Science, Oxford Brookes University, Jack Straws Lane, Marston, Oxford, OX3 OFL UK; 12 School of Experimental Psychology, University of Bristol, The Priory Road Complex, Priory Road, Clifton, Bristol, UK

**Keywords:** Quality of life, Human behaviour

## Abstract

This paper illustrates a rigorous approach to developing digital interventions using an evidence-, theory- and person-based approach. Intervention planning included a rapid scoping review that identified cancer survivors’ needs, including barriers and facilitators to intervention success. Review evidence (*N* = 49 papers) informed the intervention’s Guiding Principles, theory-based behavioural analysis and logic model. The intervention was optimised based on feedback on a prototype intervention through interviews (*N* = 96) with cancer survivors and focus groups with NHS staff and cancer charity workers (*N* = 31). Interviews with cancer survivors highlighted barriers to engagement, such as concerns about physical activity worsening fatigue. Focus groups highlighted concerns about support appointment length and how to support distressed participants. Feedback informed intervention modifications, to maximise acceptability, feasibility and likelihood of behaviour change. Our systematic method for understanding user views enabled us to anticipate and address important barriers to engagement. This methodology may be useful to others developing digital interventions.

## Introduction

The UK has one of the lowest cancer survival rates among high-income countries^1^ and quality of life (QoL) in some cancer survivors is poor, equivalent to chronic diseases.^2,3^ Problems faced include fatigue,^4^ pain,^5^ weight gain,^6^ depression and anxiety^7,8^ and fear of recurrence.^9^ Increasing physical activity, improving diet, mood management (with cognitive behavioural therapy/mindfulness) and weight loss can increase QoL in cancer survivors and may also reduce chances of recurrence.^10–15^

Existing interventions that aim to improve QoL in cancer survivors are usually delivered by healthcare practitioners (see ref. ^10^ for a review). It can be difficult to roll out clinician-based complex behaviour change interventions at scale, because in practice clinicians often lack the time or behavioural counselling skills needed to provide such support.^16^ Digital interventions offer a potential solution, as they could provide easily accessible support to large numbers.^17^ Cancer survivors have reported positive perceptions of digital interventions^17^ and emerging evidence indicates that some can be effective.^17^ In the UK, there are a lack of digital interventions for cancer survivors, which provide in-depth support to promote a wide range of cognitive and behavioural changes that could improve overall QoL (i.e. physical activity, diet, weight loss and mood management for distress and fear of recurrence).^17^ We therefore aimed to develop a digital intervention that could achieve this, named ‘Renewed’.

The planning and development of complex digital interventions is often not reported in detail, meaning that published intervention descriptions provide little detail about intervention content, how design decisions were made or how interventions are hypothesised to work, all of which are critical if the field is to build a scientific understanding of what effective interventions need to contain.^18,19^ Reviews suggest that intervention development approaches used in the development of digital interventions for cancer survivors often have a number of limitations. Reviews have concluded that there is often limited evidence of the use of theory in digital intervention design^20^ and have suggested that a lack of theoretical underpinning might be responsible for failed digital interventions for cancer survivors.^21^ A review also noted a lack of clarity about how the evidence-base informed intervention design in many interventions, making it hard to determine how interventions produced (or failed to produce) effects.^21^

It is important to consider the implementation of digital interventions for cancer survivors from the outset.^22^ In particular, to consider the environment in which an intervention might be set (e.g. National Health Service (NHS)), and how the staff who might refer cancer survivors to or assist them to use an intervention view the intervention. Review evidence shows that failure to address barriers in a healthcare environment is linked with failed digital interventions for cancer survivors, whereas those that do take the implementation setting into account (e.g. through eliciting staff views) have proven successful.^22^ Reviews have highlighted that only a minority of studies incorporate the views of stakeholders who might be crucial to implementation^22,23^ or use implementation theory that considers the environment in which an intervention will be set^22^ when developing digital interventions for cancer survivors.

Qualitative optimisation studies involving cancer survivors (or clinicians who support them) providing feedback on prototype interventions play an important role.^23,24^ Although such studies are relatively common, a review highlighted that many have small samples,^23^ meaning that saturation may not be achieved nor a wide range of views captured. Many existing studies focus on whether intervention users are satisfied with the function, usability and helpfulness of digital interventions.^23^ While understanding these components of satisfaction is useful, more focus on critical barriers to behaviour change is needed, as an inadequate understanding of such barriers may result in interventions for cancer survivors that are acceptable but ineffective.

The current paper attempts to overcome the limitations of development methodologies highlighted in reviews of digital interventions for cancer survivors. It provides a detailed report of the development process of Renewed, which used an evidence-, theory- and person-based approach to intervention planning and optimisation.^24–26^ The approach combined Patient and Public Involvement (PPI) and multi-disciplinary team input, literature reviews, theoretical modelling^27^ and iterative qualitative optimisation studies with cancer survivors and people who might support cancer survivors to use Renewed. The methodological process and findings are likely to be valuable to others developing interventions for cancer survivors.

The long-term aim is for Renewed to be made available to support all cancer survivors. However, people who have experienced different types of cancer might have different needs, and including all possible types of cancer could make it impossible to adequately power a randomised controlled trial (RCT) to evaluate Renewed. We therefore initially focussed on survivors of three common cancer types who might have varying needs, preferences and engagement with behaviour changes, across genders and ages: prostate cancer (mostly older men), breast cancer (younger and older women), and colorectal cancer (range of ages across both genders).

Below we report the planning of Renewed, followed by its optimisation with feedback from cancer survivors and staff who might support survivors in using Renewed.

## Results

### Rapid scoping review

Forty-nine studies were identified (see Tables 1 and 2). Intervention components and participant characteristics that appeared related to intervention success (or lack of success) were organised into a table of potential barriers and facilitators of intervention success (Tables 1 and 2). Findings were used to inform the Guiding Principles, Behavioural Analysis and Logic Model.

**Table 1:** Potential facilitators and barriers to intervention success based on literature review

Participant and intervention characteristics	Facilitators	Barriers
Factors influencing participation	• To regain continuity in life that was side-tracked by disease and treatment^48^ • To maintain overall health and avoid illness; to protect against recurrence^35,48–52^ • Feeling there was no other support available^53,54^	• Demographics of target group: ∘ Greater age^52,55–57^ ∘ Lower education level^55–57^ • Timing: ∘ People with more recent diagnosis took part^56,57^ ∘ Not close enough to diagnosis^53,54^ • Sense of normality: ∘ People wished to get on with their lives^58^ ∘ Did not want to assume a ‘sick’ role^58^ or be reminded of having cancer^58^
Information	• Interventions that focus on topics relevant to needs, including: ∘ Fitness/strength ∘ Energy and fatigue^35,36^ ∘ Returning to work^59^ ∘ Eating healthier^50,59–61^ ∘ Exercise/physical activity^50,52,59–64^ ∘ Financial/career concerns^59,62,65–69^ ∘ Family communication and concerns^63–65,70,71^ ∘ Dietary issues^63^ ∘ Social/domestic issues^62,67,69^ ∘ Sexual issues^62–65,67,69–73^	• Lack of knowledge/information (e.g. how to do specific exercises)^35^ • Lack of understanding how to go about starting a workout programme^36^ • Poor resources to find information and negative doctor relationship with healthcare professional^63,65,70–72^ • Current nutrition information between guidelines and health experts is conflicting^50^
Motivation, self-esteem and self-efficacy	• Belief that physical activity could assist return to normal life^36,49–51,74,75^ • Perception that being physically active is an affirmation of healthy status—desire to create distance from previous status as cancer patient^48,50^ • Not having to explain restricted movements/performance—feeling normal because limitations were allowed^36,48,76^ • Confidence that team understood issues crucial for recovery from cancer^36,74,75,77,78^ • Higher levels of coping self-efficacy: decreasing stress by viewing stressors as more manageable^79^	• Lack of confidence^35,60,66^ • Feeling embarrassed^36^ • Fear of being stared at by others in normal gyms^36^ • Lack of motivation^35,49,51^
Self-management and self-monitoring methods	• Feeling safe during exercise^36,76^ • A sense of mastery, and control over one’s body^36^ • Viewing participation in physical activity as a way of monitoring progress and achievements^48^	• Posttreatment physical symptoms and negative/persistent treatment side effects^59,62,63,65,67,68,71,73^ • Irritability/fatigue/low energy^35,59,60,62,68,73^ • Difficulty incorporating physical activity into daily routines due to comorbidities and age-related concerns^76^ • Mobility limitations^35^ • Lower ability to perform daily activities^59,62,68^ • Exercise making pain worse^35,48,80^ • Loss of a sense of control when physical symptoms have not been resolved, for example, 1 year posttreatment^77,78,81^ • Concerns about ability/skill^35^
Emotions/mood	• Humour^63,82^ • Stress management^60,62^ • Distraction^62,68,73^ • Resilience is discussed as an important aspect of healing^83^ • Acceptance/resolution^61,66,82–84^ • Improved mood and restored self-esteem^35,36^ • Physical activity may moderate unexpected emotions and fear of recurrence^57^	• Distress/sadness/fear^62,67,68,73,82^ • Depression^24,61,62,66–68,72,73,83,84^ • Anxiety/worry/preoccupation^59,61,62,65,66,69,70,82^ • Anger, frustration, resentment^64^ • Fear of recurrence during/post recovery^60,61,66^ • Feeling lost and uncertain^60–62,73,82^ • Existential/identity issues^63–65,67,68,72,73,85^ • Avoidance^61,82^ • Guilt^36^

**Table 2:** Further potential facilitators and barriers to intervention success based on literature review

Social support	• Spousal/caregiver support^63,65,86^ • Learning how others felt and experienced—realised they were not the only one^53,54^ • Perceiving supportive interaction as a morale booster^35,36,48^	• Lack of companionship^35^ • Lack of feedback or support^53,54^ • Perceiving telephone contact as too impersonal^77,78^ • Online support groups may increase helplessness, anxious preoccupation, confusion, depression at 6 months, worse QoL^21^ • Forum perceived as not useful as other’s comments not helpful^53,54^ • Not enough moderator comments in forum^53,54^
Design/content	• Theory/evidence-based content^21,60,76,79,86,87^ • Input of participants^77,78^ • Relatable: ‘everyday looking’ realistic and diverse survivor images^88^ • Simple, easy to understand format of written information^71,77,78,86,89,90^ • Convenience^53,54^ • Tailoring ∘ Age-appropriate examples^91^ ∘ Screening participants at baseline based on specific health behaviour or motivation/need to change/treatment type, as well as time from treatment completion^49,88^ ∘ Categorising text or video content into their corresponding survivorship time periods (1-2 months, 3-4 months, 5–6 months and beyond since treatment completion)^88^ ∘ Sending tailored emails^86,87^ ∘ Wanted action-oriented content^88^	• Targeting multiple behaviours may be overwhelming for participants^92^ • Lack of relatable content • Low use of role modelling video with narrative story-telling approach (may be more acceptable among minority populations)^91^ • Having a complex or ‘cold’ website layout^91^ • Individual components not standing out^91^ • Providing information perceived as ‘too much’ or ‘too difficult’^77,78^ • Not enough discussion of specific issues (e.g. erectile dysfunction)^77,78^ • Lack of tailoring to phase of illness^77,78^
Technical	• Providing emails/reminders to use programme^53,54,86,89^ • Information can be printed out^21,58,89^	• Technical/navigational difficulties^53,54,91^ • Gated parts of intervention that could not be revisited^53,54^ • Inadequate technical support^56^
Practical issues	• The opportunity to get the information needed for self-management of symptoms and problems, independent of time and location^21,55,58,87^	• Too far to travel to exercise sessions^32,93^ • Time constraints/time commitment needed^35,53,54,79,89^ • Being outdoors for exercise—being able to set the temperature, cleanliness and privacy were considered important^76^ ∘ Bad weather (restricting walking outside etc.)^35^ • Costs (gym, travel, healthy food)^35,50^ • Safety issues (walking outside in town, not safe)^35^ • Lack of equipment and adequate facilities^35^ • Daily diaries challenging to keep for some^76^

*QoL* quality of life

### Guiding Principles

Table 3 presents an overview of how the literature review informed our Guiding Principles, outlining the intervention design objectives and key intervention features that aimed to address the major challenges to engagement faced by cancer survivors.

**Table 3:** Guiding Principles for the Renewed intervention

	Literature review findings	Design objectives	Key intervention features
1	Cancer survivors might not see themselves as having health needs or as requiring an intervention and may not want to undertake healthy lifestyle changes.^49,58^	An approach which promotes wellbeing, rather than illness management	• Light in tone—Avoiding using terminology which implies illness or survivorship • Building motivation for changes from first user contacts, in recruitment materials and first session • Start by suggesting light touch/brief interventions (e.g. a few simple techniques), with options for more in-depth interventions if wanted • Allowing users to pick intervention elements and information which are most relevant to them personally
2	Cancer survivors might be sensitive to information, which implies their behaviour is inappropriate or had causal influence on their cancer.^50,85^ At the same time, if cancer survivors do not perceive the cause of a problem to be under their personal control they might feel little control or motivation to change.^49,51,77,81^	Ensure promotion of behaviour change does not stigmatise current behaviour	• Avoid arguments which could be viewed as blaming users for their cancer or poor mental health (e.g. over-promoting ‘positive coping’) • At the same time showing users the benefits of behavioural changes
3	Cancer survivors are likely to have a wide range of symptoms which affect their QoL, which would likely vary between cancer types, gender or individuals.^52^	Tailor information to be most useful, acceptable and salient to the user	• Using baseline QoL measure(s) to suggest needs/resources • For elements where the literature/our research implies it is important, we will tailor content: e.g. by gender, cancer type, QoL needs • Where we cannot easily tailor we will ask participants to select information which is most relevant to them, for instance, based on symptoms that are the most bothersome to them etc
4	Convenient access to self-management information independent of time or location could facilitate engagement^21,53–55,58,87^	Enabling easy, timely, non-intrusive access to brief information, which can be read and acted on quickly when needed	• Short sessions, where possible that the user can take something away from within a few minutes • Mobile friendly where possible (so brief amount of text on page etc) • Emails containing BCTs (so even if users only receive emails behaviour changes could be supported)
5	As the intervention was attempting to help people to improve multiple symptoms (e.g. fatigue, distress) and targeting multiple behaviours there was a risk that it might become overly large and complex, which might make the intervention overwhelming or too difficult for cancer survivors.^51,60,77^ Equally, there was a risk to the project itself in trying to develop an intervention that was too large to develop satisfactorily within the resources available	Efficient design (since many behaviours could be targeted and the intervention could become overly large, complex and expensive to develop)	• Targeting behaviours which can change multiple symptoms (e.g. physical activity which can improve fatigue, mood and general fitness) • Utilising and linking out to existing resources where possible (e.g. incorporating existing weight management and stress management interventions, linking out to existing Macmillan resources) • Strike balance between making core intervention applicable to as many cancers as possible (and cost-effective) and presenting most relevant information to ensure intervention is salient to users

*BCT* behaviour change technique, *QoL* quality of life

Challenges included ensuring the intervention would fit with users’ identities, avoid stigmatisation of current behaviours and be conveniently accessible. Users may have diverse needs and often multiple problems, which the intervention would need to support. The Guiding Principles aimed to ensure the intervention addressed all of these challenges.

### Behavioural analysis

The behavioural analysis is presented in Supplementary Table 2. The behavioural analysis shows that Renewed aims to overcome barriers to behaviour change and maximise engagement with the intervention by employing 34 behaviour change techniques and efficiently targets all 6 behavioural sources (reflective and automatic motivation, physical and psychological capability, physical and social opportunity) and 6 intervention functions (Modelling, Training, Enablement, Environmental Restructuring, Education, Persuasion) outlined in the Behaviour Change Wheel (BCW). For example, showing users the benefits of increasing physical activity (e.g. increasing energy or having better sleep) targets both psychological capability and reflective motivation. The behavioural analysis also shows that the intervention targets all four of the constructs from Normalisation Process Theory (NPT; coherence, cognitive participation, collective action and reflexive monitoring) that facilitate optimal implementation. For example, showing cancer survivors how to set diet goals enables them to self-monitor (collective action) and enabling them to review their goals on a weekly basis supports reflexive monitoring. We performed additional check of the BCW and NPT to identify any additional useful components, which might need to be considered within the behavioural analysis. We did not identify any relevant additional barriers or intervention components from the BCW or NPT.

### Logic model

**Fig. 1:**
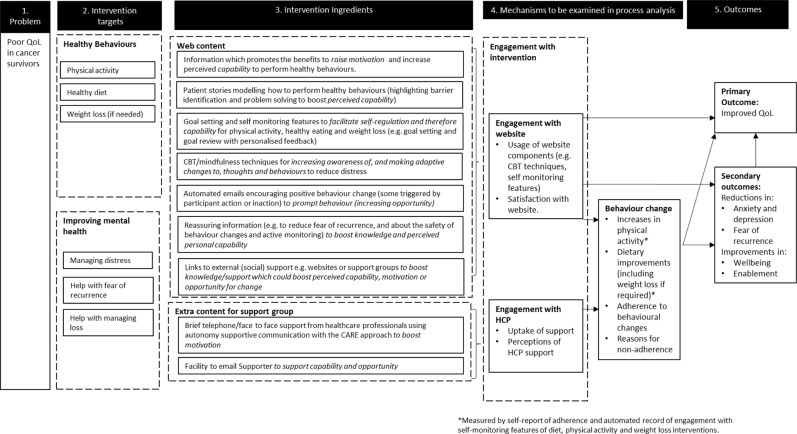
The logic model of the Renewed intervention. Starting on the left, the first column shows the problem with the intervention addresses. The second column shows the intervention targets, which are addressed in order to attempt to resolve the problem. The third column shows the intervention ingredients, which are used. The fourth column shows the mechanisms of action of the intervention, which will be later examined in process analysis. The final column shows the outcomes, which the intervention aims to impact on

Figure 1 provides an overview of the logic model, consisting of five parts: (1) The problem—poor QoL in cancer survivors. (2) Intervention targets (healthy behaviours and mental health). (3) Intervention ingredients, which incorporate the behaviour change techniques outlined in the behavioural analysis. Italics indicate the psychological construct that each intervention ingredient is targeting (e.g. perceived capability). (4) Mechanisms that will be measured in our process analysis, which are expected to influence the outcome measures either directly, or indirectly via key target behaviours. (5) Intervention outcomes.

### Optimisation study 1 with cancer survivors

Participants made many positive comments about Renewed, in particular participants often liked the look of Renewed, found the majority of the website easy to navigate and some noted that they trusted the website as it was designed by experts and found the content useful and relevant. However, a number of barriers to engagement with the Renewed digital intervention and offline behaviour changes (e.g. physical activity) were also identified. In response, intervention modifications were made to maximise engagement. Below we describe the most important of these. Supplementary Table 4 provides an overview of all the changes, including examples of participant feedback.

The biggest barrier to engaging with physical activity (in ‘Getting Active’) was the perception that activity is not possible because the participant already felt tired and that this would increase tiredness, rather than improve it. In response, we added information to acknowledging participants’ concerns, reassuring them that it can be normal to feel tired after activity, particularly in the beginning, but that this will slowly reduce and in time activity improves tiredness. We showed participants the cycle of how thinking one cannot be active because of tiredness leads to avoidance of activity, which leads to less use of muscles and more tiredness and how this cycle can be broken by gradually increasing activity. We added research evidence showing that increasing activity can help overcome cancer-related tiredness/fatigue. We discussed starting with small amounts of activity regularly, rather than larger amounts, which could take longer to recover from. Finally, we provided stories from people who had experienced extreme tiredness after cancer, which modelled how they overcame this by slowly increasing physical activity.

The most negatively perceived element of the healthy eating part of Renewed (Eat for Health) was the focus on reducing meat intake. PPI members were enthusiastic about following a healthy diet, including reducing meat consumption, but some interviewees were very negative about suggestions to opt for some meat-free meals. We therefore modified the intervention to focus on smaller changes like reducing red and processed meats, which PPI members were happy with. Our original arguments about reducing meat intake discussed not only the benefits for health but also for the planet in slowing down climate change through using less meat, as we had thought that people might find this wider benefit motivating. However, some found the discussion of helping the planet very off-putting and irrelevant to their lives. We consequently removed the statement.

### Optimisation study 2—with potential Supporters

Participants in all focus groups not only perceived benefits of helping cancer survivors to improve their QoL but also voiced concerns. Supplementary Table 5 provides an overview of the barriers to implementation raised within the focus groups with participant quotes and how we modified the intervention to address each barriers; below we discuss the most important barriers.

NHS staff in two focus groups were concerned that they were supposed to just send participants back to the website if they asked for any advice. We realised we had inadvertently included a sentence that could encourage this, which we removed. We had actually intended that Supporters would ask participants to suggest the best solution for themselves in instances where advice was requested. At the next focus group, the updated Training appeared more acceptable—staff commented that they liked the idea of the supportive nature of the questioning to enable cancer survivors to take control, rather than Supporters giving all the answers.

Volunteers at both cancer charities were concerned about only having 10-min appointments, as they usually had 30–60 min. These participants described enjoying talking and building relationships with people. They asked how to stick to 10 min if someone became distressed. We therefore added information about how to keep support appointments short but useful and steps to take if participants become distressed. Interestingly, staff at cancer charity 1 did not share volunteers’ concerns and provided reassurance to the volunteers to adjust to a new way of working. Consequently, the volunteers noted that they would just have to switch mode to follow the support protocol, but that this was achievable. This was not the case at cancer charity 2, where staff shared volunteers’ concerns. Participants from cancer charity 2 felt the support planned for Renewed was very different from their current role, which involved volunteers telling their own cancer story so that others could benefit from their experience. These participants felt unclear about what they could offer as a Supporter outside this role. Volunteers felt this made supporting Renewed less appealing as they really enjoyed forming in-depth relationships with people they were helping.

As it did not seem possible to address the concerns raised by participants at cancer charity 2, we agreed with the charity not to include them as Supporters for Renewed at this point. It appeared feasible to use staff or volunteers from the NHS or cancer charity 1.

## Discussion

This paper demonstrates a methodological approach, which could be useful to others developing digital interventions. It also extends the current literature by presenting an evidence-, theory- and person-based overview of potential barriers and facilitators to success of digital interventions, which aim to improve QoL in cancer survivors and potential intervention design solutions.

As recommended by reviews of previous digital interventions for cancer patients and survivors,^20–23^ this approach drew on the evidence-base^21^ to map out barriers to intervention success and intervention design solutions, linking these to theory that focusses on behaviour change and implementation.^20–22^ Our large qualitative optimisation studies and stakeholder (including PPI) panel involvement throughout intervention planning and development equally ensured that the views of both cancer survivors and the staff who might support them were incorporated into the design.^22,23^

We found the combination of a theory-, evidence and person-based approach particularly useful. Our theory- and evidence-based approaches (based on Medical Research Council guidance^25^) enabled us to incorporate existing knowledge into our intervention and describe it using a shared language. As our approach was not overly prescriptive^28^ it enabled rapid assimilation of existing information. Complementing this approach, the person-based approach ensured that target users’ needs were understood and accommodated to maximise engagement and implementation.^24^ Examining qualitative as well as quantitative research within our review enabled detailed insight into the needs of cancer survivors, which informed our Guiding Principles. This technique was crucial, as it stopped us from making mistakes such as discussing how lifestyle changes would help prevent cancer recurrence, which the literature showed us could have made people feel to blame for their original cancer. Equally crucial were the person-based approach qualitative optimisation studies, which enabled us to address barriers to engagement and implementation, which would have otherwise hindered the success of the intervention. An alternative theory- and evidence-based approach that could have been used is Intervention mapping,^29^ but this approach has been critiqued for being overly prescriptive and so time consuming that it is unfeasible for many developers.^28,30^ An alternative to the person-based approach could be user-centred design^31^ but this approach is often more focussed on issues of usability and navigation, with less critical focus on behaviour change, which is essential if interventions are to successfully change behaviour.^24^ The identification of behavioural issues to address and intervention elements in the behavioural analysis was also crucial. The process of mapping the behavioural analysis onto theoretical models (BCW^32^ and NPT^33^) and taxonomy of behaviour change techniques^34^ did not feed into intervention development, as this mapping did not identify important barriers or intervention components that we had not already considered based on our evidence review, qualitative work and our existing knowledge of theory. However, this process did enable us to detail the content of our intervention in a transparent way using a shared language from taxonomy and theory. The logic model was also crucial for documenting the proposed theory of action of the intervention; this model will be further refined and tested in the process evaluation. One disadvantage of adopting a theory-, evidence- and person-based approach is the amount of time it can take and some developers may find that their situation does not allow them to complete all aspects of this approach. However, this approach can be used flexibly as resources allow. For example, it may be necessary to limit the scope of the reviewing and qualitative studies and supplement them with rapid stakeholder consultation.

Some barriers to behaviour change identified within our qualitative optimisation study with cancer survivors were also identified in our rapid scoping review. For example, we knew that cancer survivors had concerns about getting more active when they were fatigued^35,36^ and had attempted to address this in Renewed, but qualitative feedback indicated that we had not done this sufficiently, prompting further modifications to address this concern. This highlights how potentially valuable qualitative optimisation studies are; even when barriers are known and teams very experienced, it is not possible to create a perfect prototype of a digital intervention.

NHS staff raised only minor concerns about the support protocol, which were easy to address with intervention modifications. Volunteers in both charities raised concerns about how the support protocol differed from their usual way of working (e.g. appointment length). Staff at charity 1 helped volunteers to make sense of the new way of working, provided reassurance and noted the benefits of brief, structured support. NPT would describe this as vital sense making work that is needed for successful implementation (named coherence in NPT^33^). The usual roles of volunteers in charity 2 involved volunteers telling their own cancer story, which differed significantly from the Renewed support and volunteers therefore could not see the value they personally would bring to supporting Renewed—in terms of NPT this was a challenge to cognitive participation and successful implementation.

While our qualitative optimisation studies enabled us to identify and address barriers to cancer survivors’ engagement with the digital intervention and behaviour changes, an important question remains as to whether using the intervention leads to cancer survivors changing their behaviour and improving their QoL. Our own work and the work of others suggests that, while there are a subgroup of cancer survivors who are motivated to engage in behavioural changes, others are not.^37,38^ Testing the effectiveness of Renewed is therefore a crucial next step and we are currently undertaking a large trial (*N* = 2500) to evaluate its effectiveness and cost-effectiveness. If successful, Renewed has the potential to be a highly accessible and cost-effective digital intervention capable of widespread implementation.

Our integrated evidence-, theory- and person-based approach enabled a systematic and rigorous approach to intervention planning. However, as limited time meant that we were only able to conduct a rapid scoping review, we limited our review to literature published over the past 20 years in 3 databases and did not search the grey literature, meaning it is possible that some literature was missed. Nevertheless, the review provided vital evidence and a detailed insight into target users’ needs and potential barriers to intervention success. While we followed the 5 core steps for rapid scoping reviews set out by Arskey and O’Malley,^39^ we did not include their sixth optional step of seeking consensus from a wide range of parties on the results of the scoping review in order to refine the findings, doing this might have helped us to refine the findings in some way, but as we had already sought feedback from our expert and PPI development group, we did not feel this step was sufficiently high priority to conduct within our limited development timeframe.

A strength of our approach was the complementary involvement of PPI and stakeholder involvement in the development team, with collection of data from a large and diverse range of cancer survivors and potential supporters. This allowed us to sample the views of people who were not represented in the development team and had views that differed in important ways.

Collecting data from cancer survivors and potential Supporters enabled us to make modifications to optimise Renewed and the Supporter Training. Our overall sample of potential supporters was large, including workers from different organisations and job roles. Men were underrepresented; although men less commonly work in these roles, it is possible male nurses or cancer charity workers might hold different views to those captured here. It might have been useful to sample from other charities. In practice, this was not possible as other charities felt unable to provide support alongside Renewed because of the resource commitment involved. In the case of both our qualitative optimisation studies, it is possible that those who participated may hold different views to those who chose not to participate.

Our approach to tabulation of feedback to inform intervention modifications was systematic, rigorous and rapid, using established criteria that guide digital intervention optimisation.^40^

In summary, Rigorous approaches to digital intervention development can ensure that new interventions have maximum chance of success and avoid wasting resources on evaluating or implementing sub-optimal interventions. This paper provides a detailed illustration of a methodological approach to intervention planning and optimisation for a digital intervention to improve QoL in cancer survivors, which may be helpful to others wanting to develop digital interventions. Our in-depth planning process highlighted barriers and facilitators to intervention success, which may be of use to other researchers and practitioners working in the field of digital medicine or cancer survivorship.

## Methods

### Overview of the intervention planning process

**Fig. 2:**
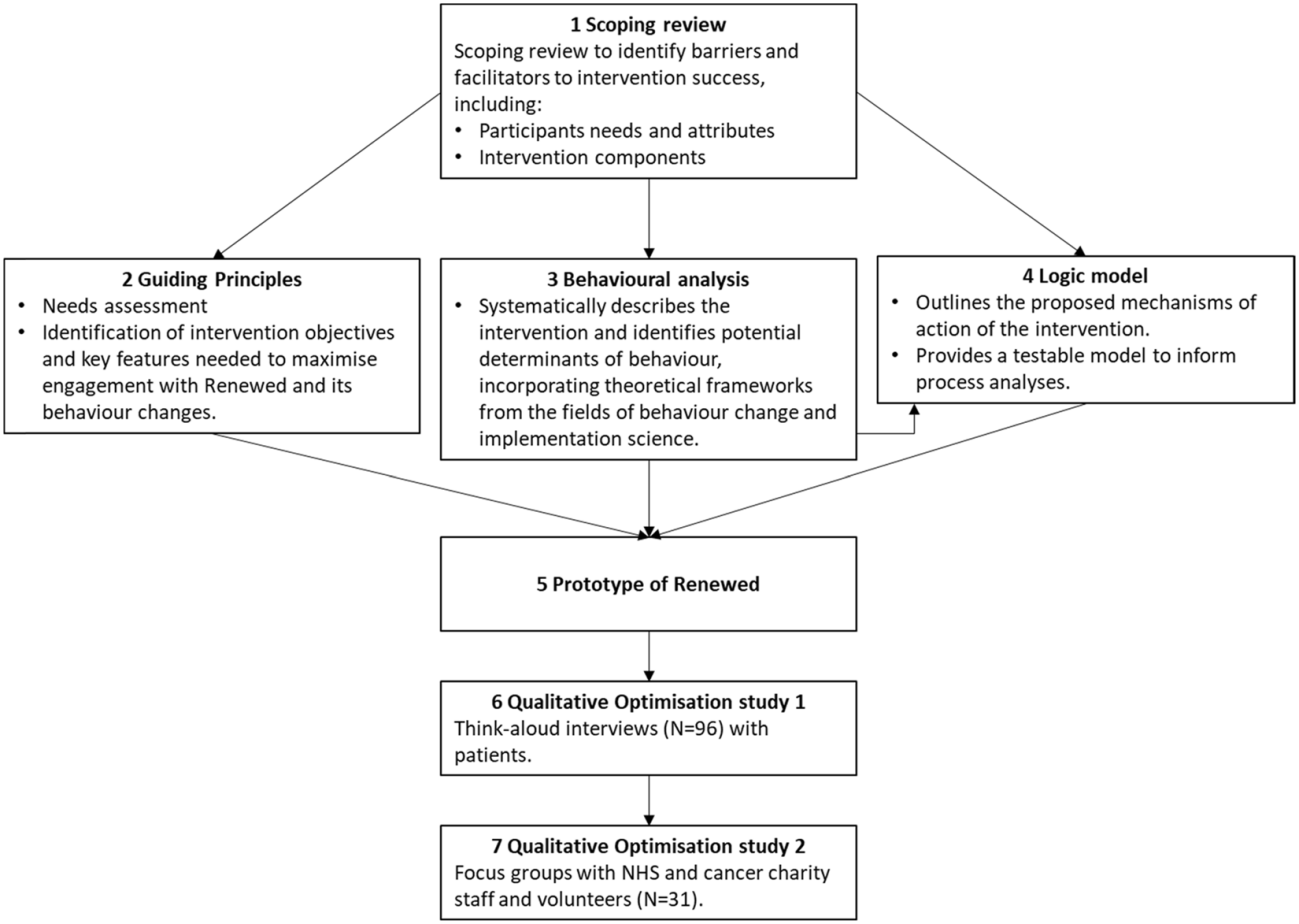
The key elements of the Renewed intervention planning process, which began with a rapid scoping review of the literature (panel 1). The results of the scoping review then informed the guiding principles (panel 2), behavioural analysis (panel 3), and logic model (panel 4). In turn, these informed the prototype of renewed (panel 5), which was then refined in two qualitative optimisation studies, the first with patients (panel 6) and the second with NHS and cancer charity workers (panel 7)

Figure 2 provides an overview of the intervention planning process, which was based on an integrated evidence-, theory- and person-based approach.^24–26^ The person-based approach draws on qualitative research with target users to ensure that interventions are grounded in a detailed understanding of the user and their psychosocial context. This enables interventions to be accessible, acceptable, persuasive and motivating.^24^

When creating Renewed, we were able to adapt two of our existing digital interventions: POWeR+ for weight management^41^ and Healthy Paths for distress management.^42^ Intervention planning for Renewed therefore focussed on creating content to support physical activity and healthy eating, plus an introduction to raise motivation and guide users in choosing which healthy changes would suit them best.

Intervention planning first drew on the existing evidence base through a *rapid scoping review* of barriers and facilitators to the success of interventions that aim to improve QoL in cancer survivors. Review findings informed the development of ‘*guiding principles*’,^24^ theory-based ‘*behavioural analysis*’^27^ and ‘*logic modelling*’*.*^43^ *Guiding principles* are part of the person-based approach to intervention planning and draw on existing evidence to identify key needs of target users’, which can be used to identify intervention components necessary to meet users’ needs.^24^ Theory-based *behavioural analysis*^27^ and *logic modelling*^43^ were employed to provide a comprehensive description of the intervention and its potential mechanisms of action.

The intervention development team included six PPI representatives who were survivors of breast, prostate or colon cancer, two experts in the area of cancer survivorship research, one expert in cancer survivorship services and research, two health psychologists, seven research psychologists, two general practitioners (GPs), one human–computer interaction researcher and a physical activity expert. Regular meetings with all members of the intervention development team (including PPI members) were used to discuss and agree the intervention plan and prototype materials.

The planning of Renewed began in December 2015 with a rapid scoping review. Searches were conducted from December 2015 to January 2016; the review findings informed the intervention’s Guiding Principles in March 2016. We began writing intervention content in April 2016 and by July 2016 we had created a prototype website. We then began optimising Renewed, which continued until the September of 2017, this began with qualitative optimisation study 1 (with patients), followed by optimisation study 2 (with healthcare practitioners and cancer charity workers), followed by final in-house testing of the website to ensure that all navigation and emails worked as intended before entering into our RCT evaluation. During the period of optimisation, we wrote and programmed all the email content for Renewed and also completed the behavioural analysis and logic model.

### Rapid scoping review

The timetable for intervention development demanded a rapid review of the literature, so a rapid scoping review was conducted.^39,44^ Rapid scoping reviews aim to efficiently map key findings in a particular area, allowing exploration of a large breadth of research, without following all the steps involved in systematic reviews, such as appraising the quality of each included study,^39^ they are therefore ideally suited to inform intervention development where a broad view of the literature is needed quickly. Our review aimed to identify potential barriers and facilitators to the success of interventions aiming to improve QoL in cancer survivors. This included literature that would provide a detailed understanding of target users’ needs. A rapid scoping review allowed the inclusion of a range of study designs (e.g. qualitative studies of cancer survivors’ experiences) that would be useful for addressing our aims, unlike traditional systematic reviews that tend to focus on RCT evidence to answer narrower questions about efficacy.^39^ We followed the five core steps set out by Arskey and O’Malley for rapid scoping reviews (identifying the research question, identifying relevant studies, study selection, charting the data and collating and reporting the results). We did not follow the optional sixth step (seeking expert consensus from various sources on the findings of the review to help refine them) as our timetable for intervention development did not allow this, although we did seek feedback from our PPI and expert development group, who were happy with our findings. We also conducted a qualitative synthesis that explored components of digital interventions for cancer survivors, which might influence uptake, acceptably, feasibility and effectiveness (reported elsewhere^17^—see Supplementary Table 1 for summary).

**Fig. 3:**
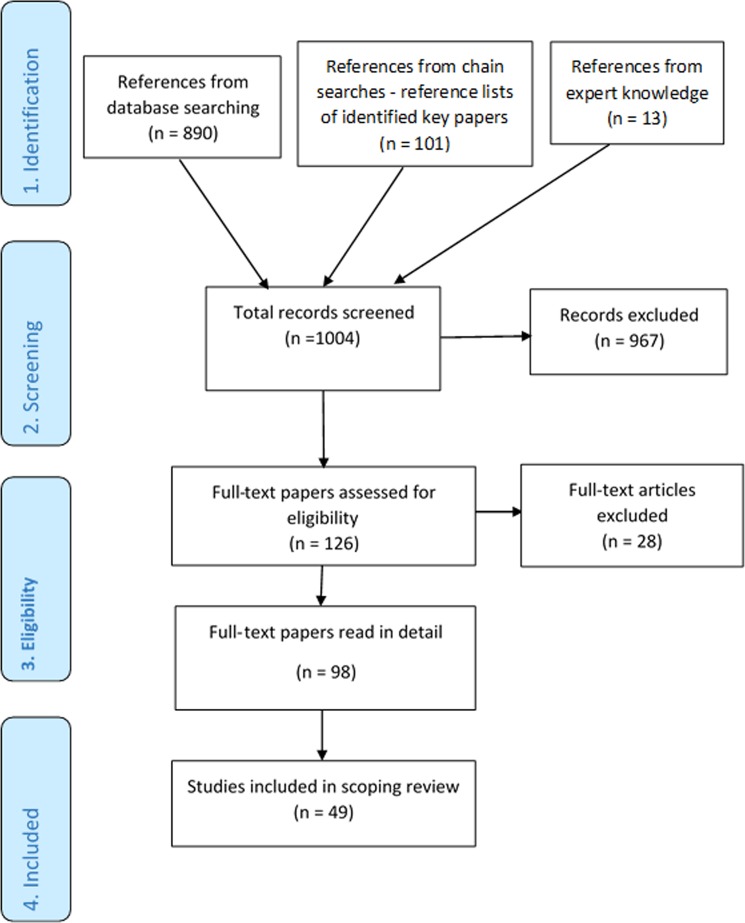
A PRISMA flow diagram for the rapid scoping review. The first row shows the identification of potentially relevant literature, the second row describes the screening and the third row shows the number of papers assessed for eligibility and the number of full text papers read in full. The fourth row shows the number of studies included

Searches were conducted in the Cochrane Library, DARE (1996–March 2015—when DARE stopped publication), Ovid MEDLINE (1996–November 2015) and PsycINFO (1996–November 2015), Box 1 outlines the search strategy and Fig. 3 provides a PRISMA flowchart. Originally, we limited searches to the past 20 years (as we had limited time). We found that more recent studies included more relevant interventions (for example, digital interventions were rare in the 90s). Further papers were identified by experts in the team and from reference lists of identified studies. We screened the search results for references that met the following criteria: qualitative or quantitative studies or reviews that reported experiences of cancer survivors or evaluations of interventions for cancer survivors who had completed primary treatment for breast, colorectal or prostate cancer, with needs relating to QoL (Fig. 1). We only read papers that were published in English, we did not review the grey literature or contact authors to search for additional papers. Data items were extracted (study date, design, intervention, potential barriers/facilitators) to a preliminary table to allow discussion between the team of barriers/factors, which could inform the Guiding Principles, Behavioural Analysis and Logic Model. A final table was produced that provided an overview of potential barriers and facilitators to the success of interventions, which aim to improve QoL in cancer survivors (see ‘Results’ section). We followed the PRISMA guidelines for reporting scoping reviews^45^ (see Supplementary Table 1 for checklist).

### Guiding principles

In line with our person-based approach, we developed brief ‘Guiding Principles’, which outlined what Renewed needed to contain in order to meet target users’ needs and maximise engagement.^24^ Drawing on our understanding of target users from our rapid scoping review, we identified key behavioural issues, needs or challenges that the intervention needed to address. We then formulated intervention ‘design objectives’ (i.e. what the intervention needed to include to meet users’ needs) and ‘key intervention features’, which intended to address each objective.

### Behavioural analysis

The behavioural analysis involved using evidence from the review and expert consultation (with the multi-disciplinary team, including PPI) to identify potential barriers to each target behaviour (physical activity, diet and using the intervention). Intervention components that would address each barrier were then selected and coded using the Taxonomy of Behaviour Change Techniques,^34^ behavioural theory (BCW^32^) and implementation theory (NPT^33^) to provide a clear description of the digital intervention and enable comparison with other interventions. The BCW is a theoretical framework that provides an overview of intervention functions used in complex interventions to target key influences on behaviour.^32^ NPT highlights factors that are necessary for an intervention to be successfully implemented.^33^ Mapping onto these theoretical frameworks also allowed us to check that we had not missed any crucial potential barriers to intervention success.

### Logic model

A logic model was developed based on findings from the rapid review and behavioural analysis, which outlined a testable model of the proposed mechanisms of action of the Renewed intervention (i.e. how the intervention is thought to work).^27,43^

### Prototype intervention

Once the intervention planning had progressed sufficiently, we built a prototype of Renewed. An introductory session was designed to build motivation for engaging with behavioural changes, after this users could access the rest of Renewed: Getting Active (to increase physical activity), Eat for Health (to support a healthy diet based on increasing fruit, vegetables and whole grains and limiting saturated fats, sugar, alcohol, red and processed meats), Healthy Paths (support with feelings of distress, loss or fear of recurrence),^42^ and POWeR+ (for weight loss^41^). Table 4 outlines the intervention content.

**Table 4:** An overview of the Renewed digital intervention for patients

Patient intervention component	Content
Introductory session	• An overview of what to expect in Renewed • Based on answers to a QoL measure (the EORTC QLQ-c30) users receive tailored, personalised feedback about how Renewed could help with each of their symptoms. For example, if users were experiencing low mood then Renewed explained how the parts of the intervention which supported improving mood (Healthy Paths) or how physical activity (Getting Active) could help to boost mood and wellbeing • Links to additional information and resources which are not provided by Renewed (e.g. financial help, community support, going back to work) • Information and reassurance about the safety and efficacy of active surveillance for prostate cancer (for men undergoing surveillance) • At the end of this introductory session, users are introduced to their homepage, where they can access all the other parts of Renewed (shown below)
Getting Active	• Promotes the benefits of increasing activity and addresses common concerns (e.g. fatigue, pain) • Suggestions of how to start gently increasing activity • A goal setting and reviewing function enables self-monitoring of physical activity
Eat for Health	• Helps people to eat a healthy diet, which is high in fruit and vegetables and low in fat, sugar, alcohol and red/processed meats • Shows people the benefits of making diet changes and addresses common concerns • Provides a traffic light list of foods • Weekly goal review enables self-monitoring of diet
Healthy Paths (and a shorter app version called ‘Healthy Mind’)	• Helps people to improve mental health, reduce stress, deal with feelings of loss and reduce fears of cancer recurrence • Uses Cognitive Behavioural Therapy (e.g. behavioural activation) and mindfulness techniques, including audio-recordings of mindfulness exercises
POWeR+ for weight loss	An evidence-based website that supports weight loss, described in detail elsewhere^41^ but in brief provides: • Low-calorie or low-carbohydrate traffic light eating plans • Explores the benefits of change and addresses common concerns • Physical activity support (walking or any other physical activity) • 25 sessions that cover topics, such as coping with cravings or relapse prevention • Weekly weight and goal review

*QoL* quality of life

As human support can boost the effects of digital interventions,^46^ we wanted to test whether providing brief support could boost the effects of Renewed. The Renewed intervention therefore also needed to include a facility for participants to contact their ‘Supporter’. Renewed users will be offered three 10-min appointments (face-to-face/by telephone) with their Supporter. Support is based on the CARE (Congratulate, Ask, Reassure, Encourage) approach (detailed elsewhere^47^), designed to boost autonomous motivation and engagement by listening to participants and helping them to decide what they want to do, rather than giving advice. We developed online training to show Supporters how to use CARE (Table 5 provides an overview).

**Table 5:** Overview of prototype online Supporter Training

Supporter Training component	Content
Introduction	• Overview of the aims of Renewed • An overview of each of the parts of Renewed that patients can access (e.g. Getting Active, Eat for Health), which explains the benefits that patients can gain from engaging in each part
Details of the Renewed RCT	• An overview of the RCT design and the study processes (e.g. follow-up) that patients will experience during the trial • Information about how much additional human support patients will be able to access from Supporters • Study inclusion/exclusion criteria
What Renewed involves for Supporters	• What the Supporter role involves—active listening, not giving advice • An introduction to the CARE (congratulate, ask, reassure, encourage) approach and how to use it • What nurses and healthcare assistants who have previously used the CARE approach to support patients. Addressing concerns about not providing advice • What patients have previously said about the CARE approach (e.g. how it makes them feel empowered) • Examples of things that Supporters have said to patients when using each of the aspects of CARE (e.g. providing reassurance) • Tips on what to do and what to avoid when implementing CARE • Practicalities of providing support, e.g. when to expect to hear from patients, how to keep a log of the support provided. This includes a flow chart of all the actions that Supporters need to take during the study • Sending supportive emails to patients who don’t request a support appointment • FAQs—covering topics like what to do if a patient requires technical support

*FAQ* frequently asked question, *RCT* randomised controlled trial

### Intervention optimisation overview

After building the prototype of Renewed and the supporter training, we took a person-based approach to intervention optimisation.^24^ This involved conducting qualitative interviews with cancer survivors to identify intervention modifications needed to maximise engagement with the intervention and behaviour change. We also conducted focus groups with potential Supporters (cancer charity and NHS workers) to explore who might be most appropriate to provide support alongside Renewed and to identify barriers to implementation. These studies are described below and the COREQ checklist for both qualitative studies can be found in Supplementary Table 3.

Ethical approvals for both qualitative optimisation studies were gained from the University of Southampton (ref no. 191936) and NHS ethics committees (ref no. 17658). Written informed consent was obtained from all individual participants included in this manuscript.

### Qualitative optimisation study 1—with cancer survivors

Thirty-two people who had completed treatment for breast, colorectal or prostate cancer in the past 10 years were recruited from GP practices in the South of England (see Table 6 for sample characteristics). Each participant took part in three qualitative think-aloud interviews, where they used Renewed while saying what they were thinking aloud. This enabled us to gauge immediate reactions to intervention content. Next, semi-structured interview questions explored what participants liked, disliked and thought should be changed within Renewed. Interviews were transcribed and used to inform intervention modifications. This process involved recording in a table all positive and negative perceptions of the intervention, to identify changes necessary to improve how acceptable, persuasive, motivating and likely to change behaviour the intervention was.^40^ When deciding whether to implement an intervention change, we considered whether each barrier was mentioned by more than one participant, whether the barrier was critical to behaviour change and whether the change was in line with Renewed’s Guiding Principles.^40^ We prioritised implementing changes that were viewed as crucial to behaviour change. Lower priority changes were made if they were quick and easy to implement. Occasionally, it was obvious that a potential intervention modification was essential even if negative feedback came from a single participant, as it would be very likely to influence behaviour change. At other times, more participant views were needed to decide whether a change was required. Potential changes were discussed within team meetings and agreed changes implemented. This was an iterative process whereby 3–5 participants were interviewed, feedback was tabulated and discussed, changes made to the intervention and then further interviews conducted. We continued this process until no further important required modifications were identified—a particular type of saturation specific to intervention development.^40^

**Table 6:** Characteristics of cancer survivors in qualitative optimisation study 1

Age in years	
Mean	68.8
Standard deviation	10.8
Range	44–90
Gender	
Male	18
Female	14
Types of cancer	
Prostate	13
Breast	11
Colon	8
Years since treatment	
Mean	3.8
Standard deviation	2.6
Range	0-9
Education level	
No education	2
Secondary School	9
College/Sixth form (postsecondary)	10
Undergraduate	4
Postgraduate	7

Table adapted from a report of the secondary analysis of this qualitative data, with permission from the authors^37^

### Qualitative optimisation study 2—with potential Supporters

Seven focus groups explored possible Supporters’ perceptions of potentially supporting cancer survivors using Renewed and the online training. Five focus groups were conducted with GP practice staff (nurses, GPs, healthcare assistants, *N* = 21; see Supplementary Box 2 for focus group schedule). Two focus groups were conducted with staff and volunteers from two cancer charities (*N* = 10) (charity names have been removed to protect the identities of the participants). Table 7 provides an overview of the participants who attended each focus group. Participant feedback was recorded in a table (as described in optimisation study 1) and informed modifications to the training; we continued until reaching saturation (as described in optimisation study 1). The data also helped us identify the most suitable supporters of Renewed to use in our trial.

**Table 7:** Focus group participant characteristics in qualitative optimisation study 2

Focus group	Female	Male
Cancer charity 1	3 volunteers, 2 staff	
Cancer charity 2	3 volunteers, 2 staff	
NHS 1	3 practice nurses, 1 assistant practice manager	
NHS 2	2 practice nurses, 1 HCA, 1 practice manager, 1 GP	
NHS 3	3 practice nurses, 1 HCA, 1 GP	
NHS 4	2 practice nurses	1 GP
NHS 5	2 practice nurses, 1 HCA, 1 practice manager	1 GP

*GP* general practitioner, *HCA* healthcare assistant, *NHS* National Health Service

Box 1 Search strategy for rapid scoping review Search strategy

• Combinations of terms for cancer or cancer survivorship, or rehabilitation (intervention or programme or self-management or self-management or health education or self-care or self-care or self-monit* or self monit* or surviv*),

• Quality of life (quality of life)

• Methodology (review or synthesis or meta-ethnography qualitative or grounded or interview or focus group* or ethnograph* or phenomenol* or view* or experience*).

• Interventions (intervention or programme or self-management or self-management or health education or self-care or self-care or self-monit* or self monit*)

• Technologies (internet or online or digital or web or e-health or computer or technolog* or telecommunication* or multimedia or PC or website or www or cellular phone or cell phone or mobile or smartphone or smart phone or electronic or ehealth or mhealth or m-health or telemedicine or text messag* or email or telehealth or teletherap* or telemonit*)

### Reporting summary

Further information on research design is available in the Nature Research Reporting Summary linked to this article.

## Supplementary information

reporting summary

Supplementary materials.

## Data Availability

Quotes from participants are in the supplementary tables; the full data sets available from the corresponding author upon reasonable request.

## Supplementary information

**Supplementary information** accompanies the paper on the *npj Digital Medicine* website (10.1038/s41746-019-0163-4).

## References

[1] Coleman M (2011). Cancer survival in Australia, Canada, Denmark, Norway, Sweden, and the UK, 1995–2007 (the International Cancer Benchmarking Partnership): an analysis of population-based cancer registry data. Lancet.

[2] Elliott J (2011). The health and well-being of cancer survivors in the UK: findings from a population-based survey. Br. J. Cancer.

[3] Department of Health – Quality Health. *Quality of Life of Cancer Survivors in England: Report on a Pilot Survey Using Patient Reported Outcome Measures (PROMS)* (DOH, London, 2012).

[4] Prue G, Rankin J, Allen J, Gracey J, Cramp F (2006). Cancer-related fatigue: a critical appraisal. Eur. J. Cancer.

[5] Green CR, Hart‐Johnson T, Loeffler DR (2011). Cancer‐related chronic pain: examining quality of life in diverse cancer survivors. Cancer.

[6] Courneya KS, Katzmarzyk PT, Bacon E (2008). Physical activity and obesity in Canadian cancer survivors. Cancer.

[7] Brown LF, Kroenke K, Theobald DE, Wu J, Tu W (2010). The association of depression and anxiety with health‐related quality of life in cancer patients with depression and/or pain. Psychooncology.

[8] Mitchell AJ, Ferguson DW, Gill J, Paul J, Symonds P (2013). Depression and anxiety in long-term cancer survivors compared with spouses and healthy controls: a systematic review and meta-analysis. Lancet Oncol..

[9] Simard S (2013). Fear of cancer recurrence in adult cancer survivors: a systematic review of quantitative studies. J. Cancer Surviv..

[10] Duncan M (2017). Review of systematic reviews of non-pharmacological interventions to improve quality of life in cancer survivors. BMJ Open.

[11] Friedenreich, C. M., Neilson, H. K., Farris, M. S. & Courneya, K. S. Physical activity and cancer outcomes: a precision medicine approach. *Clin. Cancer Res.* **22**, 4766–4775 (2016). 10.1158/1078-0432.CCR-16-0067 27407093

[12] Kassianos AP, Raats MM, Gage H, Peacock M (2015). Quality of life and dietary changes among cancer patients: a systematic review. Qual. Life Res..

[13] Mishra, S. I. et al. Exercise interventions on health-related quality of life for cancer survivors. *Cochrane Database Syst. Rev.* CD007566 (2009). 10.1002/14651858.CD007566.pub2 PMC7387117 22895961

[14] Reeves MM, Terranova CO, Eakin EG, Demark‐Wahnefried W (2014). Weight loss intervention trials in women with breast cancer: a systematic review. Obes. Rev..

[15] Schwedhelm C, Boeing H, Hoffmann G, Aleksandrova K, Schwingshackl L (2016). Effect of diet on mortality and cancer recurrence among cancer survivors: a systematic review and meta-analysis of cohort studies. Nutr. Rev..

[16] Ware LJ (2012). Exploring weight loss services in primary care and staff views on using a web-based programme. Inform. Prim. Care.

[17] Corbett T (2018). Understanding acceptability of and engagement with Web‐based interventions aiming to improve quality of life in cancer survivors: a synthesis of current research. Psychooncology.

[18] Michie S, Abraham C (2004). Interventions to change health behaviours: evidence-based or evidence-inspired?. Psychol. Health.

[19] Michie S, Abraham C (2008). Advancing the science of behaviour change: a plea for scientific reporting. Addiction.

[20] Hong Y, Pena-Purcell NC, Ory MG (2012). Outcomes of online support and resources for cancer survivors: a systematic literature review. Patient Educ. Couns..

[21] McAlpine H, Joubert L, Martin-Sanchez F, Merolli M, Drummond KJ (2015). A systematic review of types and efficacy of online interventions for cancer patients. Patient Educ. Couns..

[22] Lamort‐Bouché M (2018). Interventions developed with the Intervention Mapping protocol in the field of cancer: a systematic review. Psychooncology.

[23] Darlow S, Wen K-Y (2016). Development testing of mobile health interventions for cancer patient self-management: a review. Health Inform. J..

[24] Yardley, L., Morrison, L., Bradbury, K. & Muller, I. The person-based approach to intervention development: application to digital health-related behavior change interventions. *J. Med. Internet Res.* **17**, e30 (2015). 10.2196/jmir.4055 PMC4327440 25639757

[25] Craig P (2008). Developing and evaluating complex interventions: the new Medical Research Council guidance. BMJ.

[26] Kok, G. & Schaalma, H. in *Health**Psychology in Practice* (eds Susan, M. & Susan, M.) 203–229 (Blackwell Publishing, London, 2004).

[27] Band R (2017). Intervention planning for a digital intervention for self-management of hypertension: a theory-, evidence-and person-based approach. Implement. Sci..

[28] O’Cathain A (2019). Taxonomy of approaches to developing interventions to improve health: a systematic methods overview. Pilot Feasibility Stud..

[29] Eldredge LKB (2016). Planning Health Promotion Programs: An Intervention Mapping Approach.

[30] Hansen S, Kanning M, Lauer R, Steinacker JM, Schlicht W (2017). MAP-IT: a practical tool for planning complex behavior modification interventions. Health Promot. Pract..

[31] Erwin K (2013). Communicating The New: Methods To Shape And Accelerate Innovation.

[32] Michie S, Van Stralen MM, West R (2011). The behaviour change wheel: a new method for characterising and designing behaviour change interventions. Implement. Sci..

[33] May CR (2009). Development of a theory of implementation and integration: normalization process theory. Implement. Sci..

[34] Michie S (2013). The behavior change technique taxonomy (v1) of 93 hierarchically clustered techniques: building an international consensus for the reporting of behavior change interventions. Ann. Behav. Med..

[35] Brunet J, Taran S, Burke S, Sabiston CM (2013). A qualitative exploration of barriers and motivators to physical activity participation in women treated for breast cancer. Disabil. Rehabil..

[36] Luoma M-L (2014). Experiences of breast cancer survivors participating in a tailored exercise intervention–a qualitative study. Anticancer Res..

[37] Corbett Teresa, Cheetham Tara, Müller Andre Matthias, Slodkowska-Barabasz Joanna, Wilde Laura, Krusche Adele, Richardson Alison, Foster Claire, Watson Eila, Little Paul, Yardley Lucy, Bradbury Katherine (2018). Exploring cancer survivors' views of health behaviour change: “Where do you start, where do you stop with everything? ”. Psycho-Oncology.

[38] Hardcastle SJ (2017). A qualitative study exploring health perceptions and factors influencing participation in health behaviors in colorectal cancer survivors. Psychooncology.

[39] Arksey H, O’Malley L (2005). Scoping studies: towards a methodological framework. Int. J. Soc. Res. Methodol..

[40] Bradbury K (2018). Using the person-based approach to optimise a digital intervention for the management of hypertension. PLoS ONE.

[41] Little P (2016). An internet-based intervention with brief nurse support to manage obesity in primary care (POWeR+): a pragmatic, parallel-group, randomised controlled trial. Lancet Diabetes Endocrinol..

[42] Geraghty Adam WA, Muñoz Ricardo F, Yardley Lucy, Mc Sharry Jennifer, Little Paul, Moore Michael (2016). Developing an Unguided Internet-Delivered Intervention for Emotional Distress in Primary Care Patients: Applying Common Factor and Person-Based Approaches. JMIR Mental Health.

[43] Baxter SK (2014). Using logic model methods in systematic review synthesis: describing complex pathways in referral management interventions. BMC Med. Res. Methodol..

[44] Khangura S, Polisena J, Clifford TJ, Farrah K, Kamel C (2014). Rapid review: an emerging approach to evidence synthesis in health technology assessment. Int. J. Technol. Assess. Health Care.

[45] Tricco AC (2018). PRISMA extension for scoping reviews (PRISMA-ScR): checklist and explanation. Ann. Intern. Med..

[46] Baumeister H, Reichler L, Munzinger M, Lin J (2014). The impact of guidance on Internet-based mental health interventions—a systematic review. Internet Interv..

[47] Bradbury K (2017). Understanding how primary care practitioners perceive an online intervention for the management of hypertension. BMC Med. Inform. Decis. Mak..

[48] Midtgaard J (2015). Cancer survivors’ experience of exercise-based cancer rehabilitation–a meta-synthesis of qualitative research. Acta Oncol..

[49] Bantum, E. O. C. et al. Surviving and thriving with cancer using a Web-based health behavior change intervention: randomized controlled trial. *J. Med. Internet Res.* **16**, e54 (2014). 10.2196/jmir.3020 PMC3961702 24566820

[50] Bell K (2010). Cancer survivorship, mor(t)ality and lifestyle discourses on cancer prevention. Sociol. Health Illn..

[51] Chen Z (2015). Dissecting an online intervention for cancer survivors: four exploratory analyses of internet engagement and its effects on health status and health behaviors. Health Educ. Behav..

[52] Willems RA (2016). Cancer survivors in the first year after treatment: the prevalence and correlates of unmet needs in different domains. Psychooncology.

[53] Wootten AC (2014). Development, feasibility and usability of an online psychological intervention for men with prostate cancer: My Road Ahead. Internet Interv..

[54] Wootten AC (2014). My Road Ahead study protocol: a randomised controlled trial of an online psychological intervention for men following treatment for localised prostate cancer. BMC Cancer.

[55] Børøsund, E., Cvancarova, M., Moore, S. M., Ekstedt, M. & Ruland, C. M. Comparing effects in regular practice of e-communication and Web-based self-management support among breast cancer patients: preliminary results from a randomized controlled trial. *J. Med. Internet Res.* **16**, e295 (2014). 10.2196/jmir.3348 PMC4285721 25525672

[56] Morey MC (2009). Effects of home-based diet and exercise on functional outcomes among older, overweight long-term cancer survivors: RENEW: a randomized controlled trial. JAMA.

[57] Snyder DC (2009). Reach out to ENhancE Wellness in Older Cancer Survivors (RENEW): design, methods and recruitment challenges of a home-based exercise and diet intervention to improve physical function among long-term survivors of breast, prostate and colorectal cancer. Psychooncology.

[58] Ruland CM (2013). Effects of an internet support system to assist cancer patients in reducing symptom distress: a randomized controlled trial. Cancer Nurs..

[59] Walker Rachel, Szanton Sarah, Wenzel Jennifer (2015). Working Toward Normalcy Post-Treatment: A Qualitative Study of Older Adult Breast and Prostate Cancer Survivors. Oncology Nursing Forum.

[60] Anderson AS, Steele R, Coyle J (2013). Lifestyle issues for colorectal cancer survivors—perceived needs, beliefs and opportunities. Support. Care Cancer.

[61] Taylor C, Richardson A, Cowley S (2011). Surviving cancer treatment: an investigation of the experience of fear about, and monitoring for, recurrence in patients following treatment for colorectal cancer. Eur. J. Oncol. Nurs..

[62] Ashing-Giwa KT (2004). Understanding the breast cancer experience of women: a qualitative study of African American, Asian American, Latina and Caucasian cancer survivors. Psychooncology.

[63] Dieperink K, Wagner L, Hansen S, Hansen O (2013). Embracing life after prostate cancer. A male perspective on treatment and rehabilitation. Eur. J. Cancer Care.

[64] Staples E (2009). Men experienced and responded to the embodied and emotional effects of prostate cancer in different ways. Evid. Based Nurs..

[65] Harden Janet, Schafenacker Ann, Northouse Laurel, Mood Darlene, Smith David, Pienta Kenneth, Hussain Maha, Baranowski Karen (2002). Couples' Experiences With Prostate Cancer: Focus Group Research. Oncology Nursing Forum.

[66] Ho MY (2016). A qualitative focus group study to identify the needs of survivors of stage II and III colorectal cancer. Psychooncology.

[67] Pauwels EE, Charlier C, De Bourdeaudhuij I, Lechner L, Van Hoof E (2013). Care needs after primary breast cancer treatment. Survivors’ associated sociodemographic and medical characteristics. Psychooncology.

[68] Raque-Bogdan TL (2015). The work life and career development of young breast cancer survivors. J. Couns. Psychol..

[69] Thewes B, Butow P, Girgis A, Pendlebury S (2004). The psychosocial needs of breast cancer survivors; a qualitative study of the shared and unique needs of younger versus older survivors. Psychooncology.

[70] Hedestig O, Sandman PO, Tomic R, Widmark A (2005). Living after radical prostatectomy for localized prostate cancer. A qualitative analysis of patient narratives. Acta Oncol..

[71] Wallace M, Storms S (2007). The needs of men with prostate cancer: results of a focus group study. Appl. Nurs. Res..

[72] O’Brien R (2011). “I wish I’d told them”: a qualitative study examining the unmet psychosexual needs of prostate cancer patients during follow-up after treatment. Patient Educ. Couns..

[73] Thewes B, Butow P, Girgis A, Pendlebury S (2004). Assessment of unmet needs among survivors of breast cancer. J. Psychosoc. Oncol..

[74] Charlier C (2012). Treatment-related and psychosocial variables in explaining physical activity in women three weeks to six months post-treatment of breast cancer. Patient Educ. Couns..

[75] De Cocker K (2015). Development and usability of a computer-tailored pedometer-based physical activity advice for breast cancer survivors. Eur. J. Cancer Care.

[76] Crane-Okada R (2012). Mindful movement program for older breast cancer survivors: a pilot study. Cancer Nurs..

[77] McCaughan E, McKenna S, McSorley O, Parahoo K (2015). The experience and perceptions of men with prostate cancer and their partners of the CONNECT psychosocial intervention: a qualitative exploration. J. Adv. Nurs..

[78] McCaughan E (2013). A randomized controlled trial of a self-management psychosocial intervention for men with prostate cancer and their partners: a study protocol. J. Adv. Nurs..

[79] Carpenter KM, Stoner SA, Schmitz K, McGregor BA, Doorenbos AZ (2014). An online stress management workbook for breast cancer. J. Behav. Med..

[80] Owen JE (2005). Randomized pilot of a self-guided internet coping group for women with early-stage breast cancer. Ann. Behav. Med..

[81] Sharpley CF, Bitsika V, Christie DH (2013). Do prostate cancer patients suffer more from depressed mood or anhedonia?. Psychooncology.

[82] Wilson T, Birks Y, Alexander D (2010). A qualitative study of patient perspectives of health‐related quality of life in colorectal cancer: comparison with disease-specific evaluation tools. Colorectal Dis..

[83] McMullen CK (2008). The greatest challenges reported by long-term colorectal cancer survivors with stomas. J. Support. Oncol..

[84] Ramirez M (2010). Figuring out sex in a reconfigured body: experiences of female colorectal cancer survivors with ostomies. Women Health.

[85] Deimling GT, Bowman KF, Wagner LJ (2007). Cancer survivorship and identity among long-term survivors. Cancer Investig..

[86] Yun YH (2012). Web-based tailored education program for disease-free cancer survivors with cancer-related fatigue: a randomized controlled trial. J. Clin. Oncol..

[87] Ekstedt, M., Børøsund, E., Svenningsen, I. K. & Ruland, C. M. Reducing errors through a web-based self-management support system. *Stud. Health Technol. Informatics* **201**, 328–334 (2014). 24943563

[88] Miller SM (2015). Development and preliminary testing of PROGRESS: a Web-based education program for prostate cancer survivors transitioning from active treatment. J. Cancer Surviv..

[89] Marcus AC (2013). Cancer patient and survivor research from the cancer information service research consortium: a preview of three large randomized trials and initial lessons learned. J. Health Commun..

[90] Vallance JK, Courneya KS, Plotnikoff RC, Yasui Y, Mackey JR (2007). Randomized controlled trial of the effects of print materials and step pedometers on physical activity and quality of life in breast cancer survivors. J. Clin. Oncol..

[91] Wen KY (2012). The development and preliminary testing of a multimedia patient–provider survivorship communication module for breast cancer survivors. Patient Educ. Couns..

[92] Short CE, James EL, Stacey F, Plotnikoff RC (2013). A qualitative synthesis of trials promoting physical activity behaviour change among post-treatment breast cancer survivors. J. Cancer Surviv..

[93] Courneya KS (2007). Effects of aerobic and resistance exercise in breast cancer patients receiving adjuvant chemotherapy: a multicenter randomized controlled trial. J. Clin. Oncol..

