# Supplementary material for: Developing a digital intervention for cancer survivors: an evidence-, theory- and person-based approach
Source: NPJ Digit Med. 2019 Sep 2;2:85. doi: 10.1038/s41746-019-0163-4 (PMC6718425; doi:10.1038/s41746-019-0163-4)
Supplement: Supplementary file 2 — Supplementary materials. [file 41746_2019_163_MOESM2_ESM.pdf]

<u>Electronic Supplementary Materials</u>

Supplementary box 1: Summary of thematic synthesis

The thematic synthesis aimed to identify features of web-based interventions which might be important for cancer survivors. A systematic review was conducted. Literature was sought which related to Web-based interventions designed to improve quality of life in adults who have completed primary treatment for breast, prostate, and colorectal cancer. We included a variety of study designs (qualitative research, feasibility/pilot trials, randomized trials, and process evaluations). All available data was extracted regarding intervention characteristics, experiences, and outcomes. Textual (qualitative) data was analysed using thematic analysis. 16 papers describing 9 interventions were analysed. We identified features of web-based interventions that may improve outcomes in cancer survivors.<br><br>Four themes addressed aspects of intervention design and implementation of Web-based interventions. The themes were as follows: participant factors, characteristics of the online intervention, techniques used to change behaviour, and preferred features of Web-based interventions. These were key factors that potentially influenced a fifth analytical theme: the outcomes discussed in the papers including uptake, adherence and attrition, engagement, feasibility, efficacy, positive behaviour change, and acceptability of the interventions. Cancer survivors valued interventions that offer content specific to their fluctuating needs and are delivered at an appropriate point in the cancer trajectory. Social networking features do not always provide added benefit. Behaviour change techniques need to be employed carefully to avoid possible adverse consequences for some users.

Supplementary Table 1: Preferred Reporting Items for Systematic reviews and Meta-Analyses extension for Scoping Reviews (PRISMA-ScR) Checklist

SECTION	ITEM	PRISMA-ScR CHECKLIST ITEM	REPORTED ON PAGE #
**TITLE**			
Title	1	Identify the report as a scoping review.	N/A for this intervention planning paper
**ABSTRACT**			
Structured summary	2	Provide a structured summary that includes (as applicable): background, objectives, eligibility criteria, sources of evidence, charting methods, results, and conclusions that relate to the review questions and objectives.	The focus of this paper was on the overall approach to planning the intervention we do not include all this detail about the review in our abstract.
**INTRODUCTION**			
Rationale	3	Describe the rationale for the review in the context of what is already known. Explain why the review questions/objectives lend themselves to a scoping review approach.	The paper is about intervention planning and the review is only one part of this, so this is not done in the introduction. The rationale for the review is instead covered in the intervention planning and systematic review sections (page 4 and 5)
Objectives	4	Provide an explicit statement of the questions and objectives being addressed with reference to their key elements (e.g., population or participants, concepts, and context) or other relevant key elements used to conceptualize the review questions and/or objectives.	Aim stated on Page 5
**METHODS**			
Protocol and registration	5	Indicate whether a review protocol exists; state if and where it can be accessed (e.g., a Web address); and if available, provide registration information, including the registration number.	There was not a protocol.
Eligibility criteria	6	Specify characteristics of the sources of evidence used as eligibility criteria (e.g., years considered, language, and publication status), and provide a rationale.	Page 6
Information sources*	7	Describe all information sources in the search (e.g., databases with dates of coverage and contact with authors to identify additional sources), as well as the date the most recent search was executed.	Page 6.
Search	8	Present the full electronic search strategy for at least 1 database, including any limits used, such that it could be repeated.	See table 1
Selection of sources of evidence†	9	State the process for selecting sources of evidence (i.e., screening and eligibility) included in the scoping review.	Page 6
Data charting process‡	10	Describe the methods of charting data from the included sources of evidence (e.g., calibrated forms or forms that have been tested by the team before their use, and whether data charting was done	Page 6

SECTION	ITEM	PRISMA-ScR CHECKLIST ITEM	REPORTED ON PAGE #
		independently or in duplicate) and any processes for obtaining and confirming data from investigators.	
Data items	11	List and define all variables for which data were sought and any assumptions and simplifications made.	Page 6
Critical appraisal of individual sources of evidence§	12	If done, provide a rationale for conducting a critical appraisal of included sources of evidence; describe the methods used and how this information was used in any data synthesis (if appropriate).	Not done, as not included in the methodology we were using.
Synthesis of results	13	Describe the methods of handling and summarizing the data that were charted.	Page 6
**RESULTS**			
Selection of sources of evidence	14	Give numbers of sources of evidence screened, assessed for eligibility, and included in the review, with reasons for exclusions at each stage, ideally using a flow diagram.	Figure 2
Characteristics of sources of evidence	15	For each source of evidence, present characteristics for which data were charted and provide the citations.	Table 2
Critical appraisal within sources of evidence	16	If done, present data on critical appraisal of included sources of evidence (see item 12).	Not done
Results of individual sources of evidence	17	For each included source of evidence, present the relevant data that were charted that relate to the review questions and objectives.	Table 2
Synthesis of results	18	Summarize and/or present the charting results as they relate to the review questions and objectives.	See Table 2 which informed the intervention planning through guiding principles, behavioral analysis (pages 7-8)
**DISCUSSION**			
Summary of evidence	19	Summarize the main results (including an overview of concepts, themes, and types of evidence available), link to the review questions and objectives, and consider the relevance to key groups.	This is not as relevant to this particular paper. The discussion focuses on the whole intervention planning process which is the focus of this paper.
Limitations	20	Discuss the limitations of the scoping review process.	Page 14
Conclusions	21	Provide a general interpretation of the results with respect to the review questions and objectives, as well as potential implications and/or next steps.	Our conclusion is focused on the overall planning process which is the focus of this paper, rather than the scoping review.
**FUNDING**			
Funding	22	Describe sources of funding for the included sources of evidence, as well as sources of funding for the scoping review. Describe the role of the funders of the scoping review.	Page 16

Supplementary Table 2: Behavioural Analysis for *Renewed online* using the Behaviour Change Wheel (BCW), Normalisation Process Theory (NPT) and the Behaviour Change Techniques Taxonomy (BCTv1)

**Barriers to target behaviour**	**Intervention components**	**Target construct (BCW)**	**Intervention function (BCW)**	**Target construct (NPT)**	**Behaviour Change Technique (BCT - using 93 BCT taxonomy v1)**
	**Target behaviour: Increase in physical activity**				
Lack of time	Information about how to introduce physical activity into daily life and change small things without investing much time	Psychological Capability	Education<br>Training<br>Persuasion	Cognitive participation	4.1 Instruction on how to perform a behaviour<br>8.1 Behavioural practice/rehearsal<br>8.3 Habit formation
	Information addressing common concerns, e.g. pain, tiredness, lack of time or money when getting active;	Psychological Capability	Education	Coherence	5.1 Information about health consequences<br>5.2 Salience of consequences<br>8.2 Behaviour substitution<br>8.4 Habit reversal

Concerns about activity	Success stories of other patients demonstrating how they overcame concerns about getting more active (e.g. because of co-morbidities or fatigue)	Psychological capability; Reflective motivation; Social opportunity	Persuasion Modelling	Coherence	9.1 Credible source<br>3.1 Social support (unspecified)<br>5.6 Information about emotional consequences
	Links to other helpful websites (e.g. charity information); Recommendation to get advice from GP or practice nurse if concerned about particular health conditions; Presentation of team of experts to build credibility of the approach.	Psychological Capability; Physical capability;<br>Reflective motivation<br>Physical opportunity;<br>Social opportunity	Education; Persuasion	Collective action	9.1 Credible source<br>3.2 Social support (practical)
	Targeted information addressing concerns in specific health conditions (e.g. cancer, diabetes, Alzheimer's)	Psychological Capability	Education	Cognitive participation<br>Coherence	5.1 Information about health consequences
Lack of motivation to	Showing the benefits of increasing activity	Psychological Capability;	Education; Persuasion	Cognitive participation	5.6 Information about emotional consequences

increase physical activity		Reflective motivation		Coherence	5.3 Information about social and environmental consequences
	Regular emails to prompt increasing activity	Physical Opportunity	Environmental restructuring	Cognitive participation	7.1 Prompts/cues
	Provide guidance on how to form habits (e.g. Invite users to regularly practice physical activity in the same context (e.g. during lunch break))<br>Setting regular activity goals (i.e. type of activity, number of times)	Psychological capability;<br>Automatic motivation;<br>Reflective Motivation	Training<br>Enablement	Reflexive monitoring<br>Collective action	1.1 Goal setting (behaviour)<br>1.4 Action planning<br>4.1 Instructions on how to perform the behaviour<br>7.1 Prompts/cues<br>12.5 Adding objects to the environment<br>8.1 Behavioural practice/rehearsal<br>8.3 Habit formation
	Reviewing activity goals (including personalised feedback on progress)	Reflective Motivation	Enablement	Collective action,	1.5 Review of behaviour goal

				Reflexive monitoring	1.6 Discrepancy between current behaviour and goal<br>1.7 Review outcome goal<br>10.10 Reward (outcome)
	Advice on getting social support from family and friends	Psychological capability; Reflective motivation; Social opportunity	Environmental restructuring	Collective action	3.1 Social support (unspecified)<br>12.2 Restructuring the social environment
	Possibility to order a free activity tracker to monitor steps	Physical opportunity	Environmental restructuring; Enablement	Collective action	2.2 Feedback on behavior<br>2.3 Self-monitoring of behaviour<br>2.4 Self-monitoring of outcome of behaviour<br>12.5 Adding objects into the environment<br>8.7 Graded tasks

	Presentation of patient stories modelling overcoming barriers to motivation	Psychological capability; Reflective motivation; Social opportunity	Persuasion<br>Modelling	Cognitive participation	9.1 Credible source<br>6.2 Social comparison<br>5.6 Information about emotional consequences
Lack of knowledge/Ideas about how to increase physical activity	Presentation of various types of activities with suggestions about how to be more active (and less sedentary) in everyday life	Psychological Capability	Education<br>Training	Cognitive participation	5.1 Information about health consequences
	Presentation of patient stories modelling different ways that they increased physical activity	Psychological capability; Reflective motivation; Social opportunity	Persuasion<br>Modelling	Cognitive participation	9.1 Credible source<br>6.2 Social comparison<br>5.6 Information about emotional consequences
	**Target behaviour: Adopting a healthier diet**				
Lack of motivation to change diet	Presentation of the benefits of eating more fruit and vegetables (e.g. boosting immune system, protecting against health problems).	Psychological Capability	Education<br>Training	Coherence	5.1 Information about health consequences<br>5.6 Information about emotional consequences

	Setting weekly individual goals on healthy eating (e.g. eating more fruit and veg, eating less processed food);<br><br>Reviewing activity goals (including personalised feedback on progress): reviewing the goals and targeted feedback including health benefits; (re-) setting (new) goals; planning how to achieve the goals; provision of a print out of the individual goals	Reflective Motivation	Enablement	Collective Action, Reflexive monitoring	1.1 Goal setting (behaviour)<br>1.2 Problem solving<br>1.4 Action planning<br>1.5 Review behaviour goal<br>8.7 Graded tasks<br>2.2 Feedback on behaviour<br>2.3 Self-monitoring of behaviour<br>10.4 Social reward<br>10.9 Self-reward<br>4.1 Instruction on how to perform a behaviour<br>5.1 Information about health consequences<br>8.6 Generalisation of a target behaviour<br>4.1 Instruction on how to perform a behaviour

					12.1 Restructuring the physical environment<br>15.1 Verbal persuasion about capability<br>13.1 Identification of self as role model<br>5.6 Information about emotional consequences
	Reflection on reasons for wanting to eat more healthily (reasons are written onto a printable 'reasons to eat healthy card', which can be used as an environmental prompt to behaviour, e.g. in patients wallet).	Reflective Motivation	Persuasion	Cognitive participation	5.1 Information about health consequences<br>9.1 Credible source<br>12.1 Restructuring the physical environment
	Participant discuss stories modelling common concerns about changing diet and how they overcame these.	Reflective Motivation	Persuasion<br>Training<br>Modelling	Cognitive participation	1.2 Problem solving<br>9.1 Credible source

	Discussion of common short term side effects of changing diet (e.g. uncomfortable changes in bowel movement associated with eating more fibre) and how to address these (e.g. by increasing fibre slowly).				4.1 Instruction on how to perform a behaviour
Lack of knowledge/ ideas about how to adopt a healthier diet	Provision of weekly (printable) eating plans demonstrating healthy choices with recipes); practical tips on including more fruit and veg; links to external resources (i.e. recipes recommended for cancer patients from a cancer charity)	Reflective Motivation	Enablement Training	Collective action	4.1 Instruction on how to perform a behaviour<br>12.1 Restructuring the physical environment<br>9.1 Credible source
	Traffic light system: a guide to healthy eating Information on “red, amber, green” foods and how they relate to health print outs	Psychological Capability	Education	Coherence	4.1 Instruction on how to perform a behaviour<br>12.1 Restructuring the physical environment

					5.1 Information about health consequences<br>5.6 Information about emotional consequences
	Additional weblinks and information on drinking alcohol, weight loss, weight gain, eating problems and according sources to find further information	Psychological Capability	Education	Coherence	9.1 Credible source<br>5.1 Information about health consequences
Concerns about changing eating behaviour	Addressing of common concerns regarding the appropriateness of the eating plan for individual needs, as well as any problems regarding digestion, family, lack of energy, time until benefit, financial expenses, and various types of foods using text-based information, presentation of	Psychological Capability, Reflective motivation	Education, Persuasion	Coherence, Cognitive Participation	5.1 Information about health consequences<br>9.1 Credible source<br>1.2 Problem solving<br>4.1 Instruction on how to perform a behaviour

	research results, and stories modelling how other patients overcame these problems. .				
	**Target behaviour: Patient engagement with the intervention**				
Lack of time;<br>Forgetting;<br>Lack of motivation;<br>Other priorities	Automated email prompts<br>Regular emails to prompt behaviour change and engagement with the intervention	Physical opportunity<br>Reflective motivation	Environmental restructuring;	Cognitive participation<br>Collective action	7.1 Prompts/cues;<br>3.1 Social support (unspecified)
Lack of motivation and Perceived lack of support	Meet the team section with description of experts involved in developing the intervention to boost credibility of the intervention<br><br>Reassurance there will be support provision<br>'Contact my supporter' function	Reflective motivation<br>Psychological capability;<br>Physical opportunity	Persuasion<br>Enablement	Coherence<br>Collective action	9.1 Credible source<br>3.1 Social support (unspecified)<br>3.2 Social support (practical)<br>9.1 Credible source

	Provide a rationale for the importance of changing behaviour.<br>Use of autonomy supportive language	Psychological capability<br>Automatic motivation;	Enablement<br>Education<br>Persuasion	Coherence<br>Cognitive participation<br>Collective action	5.1 Information about health consequences<br>5.6 Information about emotional consequences<br>15.1 Verbal persuasion about capability<br>10.4 Social reward<br>10.9 Self-reward<br>15.3. Focus on past success
	Link out to other helpful websites (links to charity information);<br>Provide advice on when user may need to contact healthcare providers	Physical opportunity<br>Social opportunity	Enablement<br>Education	Cognitive participation<br>Collective action	9.1 Credible source<br>3.1 Social support (unspecified)

Supplementary table 3: Consolidated criteria for reporting qualitative studies (COREQ): 32-item checklist for qualitative optimisation studies 1 and 2

1	Which researcher/s conducted the interviews	Qualitative optimisation study 1: TCB, LP and 5 MSc students<br><br>Qualitative Optimisation study 2: KB, MS, TC
2	What were the researcher's credentials?	Qualtiative Optimisation study 1: TCB=PhD, LP= MSc in Health Psychology, the 5 MSc students had undergraduate psychology degrees.<br><br>Qualtiative Optimisation study 2: All interviewers hold PhDs.
3	What was their occupation at the time of the study?	All staff were research fellows. Students were studying for an MSc in Clinical/Health Psychology.
4	Was the researcher Male or Female?	One male student (study 1), the remainder were female
5	What experience/training did the researchers have?	All staff had experience of conducting qualitative research. All students were given detailed training in conducting think-aloud and semi-structured interviews by KB (an experienced qualitative researcher).
6	Was a relationship with participants established prior to the research?	No participants were known prior to the interviews/focus groups.
7	What did the participant know about the researcher?	Participants were told that the interviewer was a member of staff/student at the University of

		Southampton in the Psychology department and were really interested in what the participant thought of the intervention, they were told that there were no right or wrong answers.
8	What characteristics can you report about the interviewer/facilitator? e.g. *Bias, assumptions, reasons and interests in the research topic*	Qualitative Optimisation study 1: Five of the interviewers were students who were interested in the topic simply to complete a dissertation. The two members of staff who conducted the interviews were interested in intervention development of digital interventions.<br>Qualitative optimisation study 2: The 3 interviewers were interested in intervention development and behavior change. One interviewer (TC) had focused on the area of cancer survivorship for her PhD.
9	What methodological orientation was stated to underpin the study?	Pragmatic intervention development
10	How were participants selected?	Qualitative Optimisation study 1: Purposive sampling, looking to sample people with breast/colorectal/prostate cancer.<br>Qualitative optimisation tudy 2: Purposive sampling, a mix of GP practice staff and cancer charity workers.
11	How were participants approached?	Qualitative optimisation study 1: GP practices sent letters to participants. Some participants were recruited via email advertisements from cancer

		charities. Participants indicated which cancer type they had experienced and some brief demographic information on the reply slip which they completed to participate in the study.<br>Qualitative optimisation tudy 2: GP practices were recruited by the CRN and the study advertised with staff via an email from the practice manager. Cancer charities were approached by the research team, who invited their staff and volunteers via email.
12	How many participants were in the study?	Qualitative optimisation study 1: 32<br>Qualitative optimisation study 2: 31
13	How many people refused to participate or dropped out? Reasons?	Qualitative optimisation study 1: Five cancer survivors contacted us to refuse to participate because they were not interested in taking part in research. No participants dropped out.<br>Qualitative optimisation study 2: We are not aware of any refusals to participate, participants were recruited via practice managers or cancer charity staff who may not have reported refusals to us. No participants dropped out.
14	Where was the data collected?	Qualitative optimisation study 1: Most of the data was collected in participants' homes, 5 people were interviewed at the University of Southampton.

		Qualitative optimisation study 2: Focus groups took part in GP surgeries or cancer charities.
15	Was anyone else present besides the participants and researchers?	Qualitative optimisation study 1: Two participants chose to bring their partners along, who did not speak during the interview.<br>Qualitative optimisation study 2: No one else was present.
16	What are the important characteristics of the sample?	Qualitative optimisation study 1: Cancer survivors who have finished primary treatment for breast, colorectal or prostate cancer. Full details of the sample are reported in Table 1.<br>Qualitative optimisation study 2: Important characteristics include implementation setting (NHS/cancer charity) and job role. An overview of these is provided in Table 2.
17	Were questions, prompts, guides provided by the authors? Was it pilot tested?	Qualitative optimisation study 1: Yes, an overview of the questions asked is provided in the methods. This was piloted on our 6 PPI representatives, plus has been used in many other studies previously.<br>Qualitative optimisation study 2: Yes, see Appendix 9 for focus group schedule, these were not pilot tested, but were based on questions we had used in similar previous studies.

18	Were repeat interviews carried out? If yes, how many?	Qualitative optimisation study 1: Yes, 3 interviews with each participant.<br>Qualitative optimisation study 2: No, just a one off focus group
19	Did the research use audio or visual recording to collect the data?	Yes, audio recording
20	Were field notes made during and/or after the interview or focus group?	Yes
21	What was the duration of the interviews or focus group?	Qualitative optimisation Study 1: range: 26-151.47 minutes (median: 58.48 minutes)<br>Qualitative optimisation Study 2: range: 23.27 minutes -1 hour 9.50 minutes (median: 30:50 minutes)
22	Was data saturation discussed?	Yes, data saturation was achieved in both studies.
23	Were transcripts returned to participants for comment and/or correction?	No
24	How many data coders coded the data?	Qualitative optimisation study 1: 7 coders<br>Qualitative optimisation study 2: 2 coders
25	Did authors provide a description of the coding tree?	A coding tree was not produced as this was not relevant to the analysis, instead data tables were created (see methods for description).
26	Were themes identified in advance or derived from the data?	This was an inductive qualitative study identifying findings based on the data. Themes were not identified

		as this was not the purpose of this intervention development analysis.
27	What software, if applicable, was used to manage the data?	Data tabulation was conducted in Microsoft Word
28	Did participants provide feedback on the findings?	No
29	Were participant quotations presented to illustrate the themes / findings?	Yes, quotes are presented in the appendix 8 for qualitative optimisation study 1 and appendix 10 for qualitative optimisation study 2.
30	Was there consistency between the data presented and the findings?	Yes – see appendix 8 and 10 for full overview.
31	Were major themes clearly presented in the findings?	No, themes are not applicable to this intervention development approach.
32	Is there a description of diverse cases or discussion of minor themes?	No, this was not relevant to this study which did not focus on generating themes.

Supplementary table 4: Barriers to cancer survivors' engagement and intervention modifications

Participant feedback	*Participant quote*	Intervention modifications
Introductory session		
Some participants were confused by the names of the different parts of the intervention (e.g. Eat for Health, Healthy Paths) and didn't know what they would be about.	*Healthy paths… What does that mean?*	Changed the button names from intervention names to a description of the intervention (e.g. 'Eat for Health' to 'Healthy eating')
Participants raised concerns about being able to increase physical activity because of various health conditions (e.g. pain).	*I'm getting a fair bit of pain from my knees...and my legs. So, 'increase activity', I feel, is not possible because, you know, my knees and my legs won't allow me.*	A new page was added which addressed these concerns, explaining the safety and benefits of gently increasing physical activity when experiencing health problems. New pages provided further detail on how to safely increase physical activity when experiencing painful conditions and other health problems.
Eat for Health		
Participants found discussion of how reducing meat intake could help the environment off-putting.	*"I'm really sorry but I couldn't give a stuff…it's of no relevance to me whatsoever and you've completely lost me. 'It's not just good for you, it's good for the whole planet' – you're going in a completely different direction here. This is propaganda for Greenpeace or whoever you want to be and you've lost me as an old person."*	We consequently removed any mention of this.
Some participants were concerned about whether eating less meat and more vegetables would reduce their energy.	*"I mean, we eat meat for energy, don't we.* I don't feel vegetables give you any energy."	We added reassurance that a range of foods provide the body with energy, not just meat.
Some participants were concerned that the eating plan was not specifically designed for people who had cancer.	*It's not specifically for somebody who's had cancer.*	We added explanation that this eating plan is designed for people who have experienced cancer, based on the latest evidence.

Some participants discussed wanting more information about what meals to make, some wanted to see recipes or meal plans.	*What I'm really interested about for me personally, is what options they give you instead of meat, because I have a family who are very a meat and two veg family, so it's be nice to have some alternatives.*	Added a range of recipes and meal plans to show examples of how to eat a healthy diet.
Some participants perceived healthy food to be expensive.	*Unfortunately, for a lot of people, (healthy eating is) also expensive.*	A new click through page addressed this concern, showing research which looked at buying healthy and unhealthy foods from a range of supermarkets and the small cost differences between these. Information about the cost-savings of switching to budget supermarkets was included, as well as tips about eating healthily on a budget.
Some participants wondered whether they could trust links to additional information on other webistes, as they perceived that the media provide contradictory information and so it's hard to know whether information can be trusted.	*(I'm) skeptical about all this because one day we're told one thing in the press and media and the next day we're told something else*	We added a statement acknowledging concerns that information in the media can sometimes seem contradictory which also provided reassurance that the links provided came from credible sources which could be trusted as they were based on evidence.
Some older participants felt that the examples of fruit and vegetables given in patient stories on the website were unlikely to be eaten by older people.	*Why choose a kiwi fruit? That's not a fruit that many people of – again, it's a generic site, but I'm here to talk about old buggers with cancer of their prostate. They're never going to go and buy a kiwi fruit. Very few of them, anyway, that's just me... But why not mention other green things like cabbage? Broccoli? Green beans is what will people will buy.*	A wider range of fruit and vegetables were added to examples throughout Eat for Health, including the things that older people told us they like to eat.
A couple of participants were concerned that healthy meals are more complicated to make	*That said, especially because I'm cooking for a family, I don't want complicated. I don't want things that are gonna take three hours to cook or have*	We added an extra optional page which discussed how healthy meals do not have to be complicated. We also added simple to

	ingredients that you can only find in a local health shop and you can't get in Tescos.	make recipes and meal plans, plus links to further simple recipes on the internet.
Getting Active		
Perception that physical activity is not possible because the participant is already tired.	*I can't. I can't be active if I'm feeling tired, weak, and low on energy.*<br>*What I find is if I have a busy day or an active day the day after I really suffer, and I am ridiculously tired, more so than normal.*	We added reassurance that it can be tiring to increase activity (especially at first), but that this reduces with time. We added an overview of the fatigue cycle and how this can be broken by increasing activity. We added research evidence about activity improving fatigue and information about avoiding extremes of activity. We also added patient stories modelling overcoming fatigue by increasing physical activity.
Originally we presented 'Activities that you can do with family', but some participants felt did not have close family, or felt that their family would not be interested in being active with them.	*This doesn't apply to me, cos I don't have a family*	We renamed this page 'Activities that you can do with family and friends'
Some participants voiced concerns about whether they might overdo activity if they were to increase it	*It's knowing when you've overdone it, in't it? 'Cause you can either think 'Oh, yeah, this is alright.' You know, 'I'm feeling the pain' sort of thing but you may be doing more damage than good*	We added information empathising that this is a common concern and explaining how to increase activity slowly to avoid consequences of overdoing activity. Throughout the intervention we also edited other messages about increasing activity to ensure they said to do this 'slowly'.
Participants found some of the stories in Getting Active unconvincing because they perceived that a) the person in the stories was already too active, b) that they were not a cancer survivor, so would not be experiencing fatigue.	*See, she's actually got a pretty busy day anyway. And then rushing out to look after her mum. Now, you could argue that she's pretty active anyway… The other argument here - Sally hasn't had cancer has she? I have. I'm not feeling –*	We edited the stories to make them seem less active and more relatable and made it clear that they were written about people who had experienced cancer.

	you know, I'm feeling tired for a different reason.	
Originally we had assumed that participants would know how to be active (I.e. walking), but might be lacking in motivation to get more active. Initially we therefore provided little information on exactly how to get more active, but participants felt they weren't sure how to increase activity and asked for examples of what they could do.	*I'd probably like to see a little bit more, because I understand that they're good for you, but to see what the recommendations are in terms of activity breaks from sitting.*	We added examples of the kind of physical activity that people could do to increase activity and reduce sedentary time.

Supplementary box 2: Focus group schedule

- How do you currently support patients who have had cancer and finished treatment in the past?
  -What is your usual practice?

- Can you tell me a bit about what you think of the prospect of an online tool to help support patients after cancer treatment?

- So you’ve had chance to look through the online training for Supporter’s who will be doing the study (the Supporter’s Guide). Overall, what did you think of the Supporter’s Guide?
  - Can you tell me about anything that you liked about it?
  - Can you tell me about anything that you disliked about it?
  - Can you tell me about anything that you think should be changed?

- What did you think of the CARE approach- Congratulate, ask, reassure and encourage? (***take with you the materials here to easily refer to an example***).
  - Can you tell me about anything that you liked about the CARE approach?
  - Can you tell me about anything that you disliked about it?
  - IF they raise barriers to use ask: Can you tell me about any suggestions for how you might overcome that problem?

- If you were asked to provide support to patients who were using the Renewed website, how do you think you would feel about following the support outlined in the Supporter’s guide?

- How might the programme fit with your usual role?

- It would be good to hear your thoughts about implementing the intervention in everyday practice:

  - Can you tell me about the benefits you can see in implementing it?

  - Can you tell me about the any problems you can see in implementing it?

- Could you tell me about any suggestions you have to overcome these problems?

- In the study we are anticipating that patients could email Supporters through Renewed online (it would hide the Supporter's email address) to book an appointment. Then at the time of the appointment you would phone the patient to give them support. If for a moment you imagine you are a Supporter in this study, what do you think of that procedure?
    - Can you tell me about any problems that you anticipate in using that procedure?
    - Can you tell me about any suggestions you have of how we might overcome that problem?

- Still imagining that you are a Supporter in the study: If patients don't get in contact about booking a support appointment with you within two weeks of the time when they were promoted to do so then Renewed would send you an email to ask you to email the patient to send them some encouragement.
    - How would you feel about sending these emails?
    - Can you tell me about any benefits that you anticipate in sending emails to patients who don't get in touch?
    - Can you tell me about any problems that you anticipate in sending emails to patients?
    - Can you tell me about any suggestions that you have of how we might overcome this problem?

Supplementary table 5: Concerns raised in focus groups and intervention modifications

Participant feedback	Participant Quote	Intervention modification
NHS staff were concerned about not giving advice and perceived they were being asked to just send patients back to the website instead.	*It'd be quite difficult not to advise… (patients) come to expect advice from us…I think I called it stonewalling didn't I , you just, it's the message seemed to be from the (training) that we would constantly refer them back to the website and that's an approach that we certainly don't use at the moment, and that would be a big change.*	We improved the clarity of the instructions to show that if patients asked for advice (e.g. which physical activity to try) then the Supporter should put the question back to the patient, to see what they think is best for them.
NHS staff felt that healthcare assistants might be better supporters than nurses as the role didn't require medical knowledge and healthcare assistants would be capable of providing the support.	*Well you could do it with healthcare assistants. Because it's just re-enforcing ... Rather than we (nurses) telling them or advising anything…anybody can (discuss this), even without having medical knowledge, because you don't need medical knowledge.*	We decided to recruit healthcare assistants as Supporters within our trial (but would allow nurses to still conduct this role if the GP practice preferred).
Cancer charity volunteers were concerned about not building a personal relationship with the people they were supporting because support would be done remotely, or because it would be brief.	*I wouldn't like it to interfere with the face-to-face, you know that is the most important thing to me, you know as a volunteer here is actually you know meeting a person when they first come in... and that's quite important for me really.*	Staff at cancer charity 1 reassured volunteers that they would still be able to provide other kinds of support where more in depth relationships were possible, and explained the benefits of this kind of support for people who don't access such services. Volunteers seemed reassured by this so we did not make any changes to the Supporter Training.
Cancer charity volunteers were concerned about only having 10 minutes to talk to patients, as they usually had much longer. Some were worried about what to do if patients became distressed during this time.	*I did think we might struggle with ten minutes.*<br>*(If) they're actually literally breaking down on the phone … then what could we do after that? (Usually we would say we'll) pop in and I'll see you later, but apparently you can't.*	We added information about how to stick to 10 minutes within support phone calls (e.g. how to set up and end the phone call). We also added more information about what to do if a patient becomes distressed.

Volunteers from the second cancer charity were concerned that the support was very different to the support they were used to providing, which involved telling their personal cancer story. They were therefore not sure whether this support would be right for them, or what they would offer as a supporter.	*I speak to people who are having the same sort of treatment path or facing similar concerns that I had during my treatment, so I know what my added value is, as a volunteer. (In Renewed) I'm not so sure, like it isn't apparent to me what I could bring to the table, if you like*	No change was implemented.
NHS and cancer charity staff wanted to be able to access what patients could see in Renewed online as they were concerned that they might not know enough about the programme otherwise.	*If you've never seen it or, it's very hard to support, not knowing any more about it.*	We added links to the content of Renewed online at the end of the Supporter's training.
Originally, the supporter training discussed how Renewed online aimed to improve QoL and prevent cancer recurrence. However, some NHS staff were concerned about this because they were worried it might make some supporters give assurances to patients that if they followed all the advice on Renewed that they would definitely not get cancer again.	*It's almost offering perhaps too much assurance that you're doing everything right, you're doing, you know you're doing the best you can, you're not going to get cancer, and I just thought, is there a danger here that you give false assurances to someone?*	We rephrased 'prevent cancer recurrence' to 'lower chances of recurrence' throughout the Supporters training.
Some NHS and cancer charity staff wanted emails from cancer survivors to be sent to a central email address (at their GP practice, or branch of their cancer charity), just in case the supporter was away and unable to respond quickly	*If someone is off on leave or if they're unexpectedly sick, or they're part-time, they're not going to get it, so we would tend to use a central email address within the practice.*	We enabled Supporters to be able to sign up a central email address, as well as their own, so that both could receive emails. We also added explanation to the training website that patients had been told that the supporter might not always be able to respond quickly.
Participants from cancer charity 2 wanted to be able to request clinical supervision, which they were used to receiving if they were uncomfortable or distressed during providing Support to cancer survivors (the nature of their usual support calls was much more in depth discussion of how people were feeling	*It can be very hard to listen from a deep and dark place if they're feeling that for instance. It's only…what you would do with that as a supporter.*<br>*Would there be some support for (Supporters), by telephone, should (Supporters) come across anything that*	As the calls are not meant to be therapy, are likely to be short and follow the CARE approach (which keeps people focussed on the behaviour changes that they are trying out) it is very unlikely that people would discuss things that might require clinical supervision. The trial team were unable to

than is used in CARE, with calls lasting for 30-60 minutes)	*makes them feel uncomfortable or out of their depth*	provide regular clinical supervision to Supporters. So it was agreed that a protocol change would not be made in this instance.
NHS and charity staff and volunteers wanted to know how many cancer survivors they might be asked to support.	*I would want to know you know how many participants you're expecting to have you know, … that's okay if you've only got ten patients over however long, but if you're expecting a hundred.*	We added information to explain how many patients Supporters could expect to support (approx. 5-10 per GP practice, most Supporters would only be supporting one GP practice).
Some NHS staff wanted to be able to share a Supporter role with a second member of staff who could take over if they were unavailable for more than a couple of weeks.	*The other thing that would be handy is if they, if you have got a nurse who's a supporter, that they have a buddy, because obviously sickness happens, holiday happens, and what you don't want is that patient kind of getting lost in the ether because their nurse is on leave for three weeks or their nurse has gone on long-term sick for six weeks.*	We enabled Supporters to nominate a second person who could complete the online training and take over as a Supporter if required.
Some participants were concerned about patients having their personal email address	*I personally wouldn't want patients having my email address.*	We added reassurance to the online training to state the patients would not be able to see the Supporters email address as Renewed online is able to hide this.
Some NHS staff were concerned about the 'congratulate' part of the CARE model, as they perceived they would need to congratulate patients in not succeeding in their goals, which was not the case.	*It's more difficult to say well done, well done… they've put on another three kilos, well done. Well done for that, you know.*	We added information about how to use the CARE approach when patients are not succeeding with their chosen changes.
